# Ectopic Expression Screen Identifies Genes Affecting *Drosophila* Mesoderm Development Including the HSPG Trol

**DOI:** 10.1534/g3.114.015891

**Published:** 2014-12-23

**Authors:** Nathanie Trisnadi, Angelike Stathopoulos

**Affiliations:** Division of Biology and Biological Engineering, California Institute of Technology, 1200 East California Boulevard, MC 114-96, Pasadena, California 91125

**Keywords:** *Drosophila*, embryogenesis, Trol Syndecan, fibroblast growth factors, heparan sulfate proteoglycan, mesoderm cell migration

## Abstract

Gastrulation of the embryo involves coordinate cell movements likely supported by multiple signaling pathways, adhesion molecules, and extracellular matrix components. Fibroblast growth factors (FGFs) have a major role in *Drosophila melanogaster* mesoderm migration; however, few other inputs are known and the mechanism supporting cell movement is unclear. To provide insight, we performed an ectopic expression screen to identify secreted or membrane-associated molecules that act to support mesoderm migration. Twenty-four UAS insertions were identified that cause lethality when expressed in either the mesoderm (Twi-Gal4) or the ectoderm (69B-Gal4). The list was narrowed to a subset of 10 genes that were shown to exhibit loss-of-function mutant phenotypes specifically affecting mesoderm migration. These include the FGF ligand Pyramus, α-integrins, E-cadherin, Cueball, EGFR, JAK/STAT signaling components, as well as the heparan sulfate proteoglycan (HSPG) Terribly reduced optic lobes (Trol). Trol encodes the ortholog of mammalian HSPG Perlecan, a demonstrated FGF signaling cofactor. Here, we examine the role of Trol in *Drosophila* mesoderm migration and compare and contrast its role with that of Syndecan (Sdc), another HSPG previously implicated in this process. Embryos mutant for Trol or Sdc were obtained and analyzed. Our data support the view that both HSPGs function to support FGF-dependent processes in the early embryo as they share phenotypes with FGF mutants: Trol in terms of effects on mesoderm migration and caudal visceral mesoderm (CVM) migration and Sdc in terms of dorsal mesoderm specification. The differential roles uncovered for these two HSPGs suggest that HSPG cofactor choice may modify FGF-signaling outputs.

Embryonic development requires integration of multiple complex processes such as cell movement, proliferation, and differentiation, all of which are regulated by signaling pathways. Therefore, to ensure proper execution of the first movements during embryonic development that encompass the process of gastrulation, for instance, it is important that signaling pathway activation is tightly regulated (Solnica-Krezel and Sepich 2012). In *Drosophila*, the embryo undergoes extensive cell movements during gastrulation that support its lengthening through the process of germ-band elongation, as well as the establishment of a multilayered state through invagination of the mesoderm in ventral regions and its subsequent migration, internally, along the inner side of the ectoderm.

Fibroblast growth factor (FGF) signaling is important in supporting mesoderm migration during gastrulation of the *Drosophila* embryo. The *Drosophila* FGFs Pyramus (Pyr) and Thisbe (Ths) and their receptor Heartless (Htl) have been previously shown to function in supporting this process (Winklbauer and Muller 2011; Bae *et al.* 2012). FGF signaling regulates the collective migration of the mesoderm because in mutants two populations of cells can be defined: cells in contact with the ectoderm move in a uniformly directional manner, whereas those located at a distance move aberrantly without apparent direction. The roles of FGF in this process include guiding symmetrical collapse of the invaginated tube of mesoderm cells as well as supporting formation of a monolayer of cells at the end of the migration process. Both these movements guide cells in the radial direction, and similar phenotypes (at least in part) were identified for the Rap1 GTPase and β-PS integrin, Myospheroid (Mys) (McMahon *et al.* 2008, 2010). Rap1 mutants exhibit collapse defects, whereas in both Rap1 and Mys mutants cells fail to intercalate and do not form a monolayer. Because a subset of mesoderm cells is able to spread dorsally in these mutants (McMahon *et al.* 2008), other inputs besides FGF, Rap1, and Mys are also likely important for guiding directional movement of mesoderm cells during gastrulation.

Specifically, we hypothesized that additional signaling pathways and/or regulators of cell adhesion may act to support mesoderm migration at gastrulation. To investigate how cells were able to migrate in the absence of FGF signaling and also to discover additional components in the FGF pathway, we conducted a screen of a collection of UAS insertions located near cell-surface or secreted (CSS) proteins first used in a neuronal pathfinding screen (Kurusu *et al.* 2008). The UAS/GAL4 system was used to ectopically express candidate genes in either the presumptive mesodermal or the ectodermal tissues. We postulated that important signals guiding this process normally would be differentially expressed in tissues in the embryo, either in the mesoderm or in the ectoderm, to provide positional information to guide mesoderm cell movements. In this way, using this CSS collection, we identified 24 genes, of 311 tested, that impact *Drosophila* development when ectopically expressed; 10 of which were subsequently shown to specifically affect *Drosophila* gastrulation when mutated. We focused analysis on one gene isolated in this screen encoding a heparan sulfate proteoglycan (HSPG), Terribly reduced optic lobes (Trol), due to previous research linking HSPGs to FGF signaling. Crystal structures have revealed that HSPGs bind to the FGF ligand and receptor as a heterotrimeric complex (*i.e.*, FGF-HSPG-FGFR) (Pellegrini *et al.* 2000). It has been proposed that HSPGs facilitate ligand–receptor interaction and/or stabilize the FGF-FGFR dimer complex (Ornitz 2000).

HSPGs comprise a core protein attached with highly modified heparan sulfate glycosaminoglycan side chains that provide specificity towards the regulation multiple signaling pathways during development (Lin 2004). There are only four known core proteins in *Drosophila*: transmembrane Syndecan (Sdc); two membrane-anchored glypicans Dally and Dally-like (Dlp); and the extracellular matrix protein Trol. Trol is the homolog of mammalian Perlecan (Pcan), and several lines of evidence support the view that Pcan promotes multiple pathways including FGF signaling in vertebrates (Farach-Carson *et al.* 2014). For instance, *in vitro* experiments measured a gradient of FGF-2 and correlated its levels with Pcan and pERK, a signal measuring activation of the Ras intracellular signaling pathway downstream of FGFR activation (Wu *et al.* 2014). Studies in the developing mouse heart show specific Pcan modifications (*i.e.*, sulfations) are required for binding of different FGF-FGFR complexes (Allen and Rapraeger 2003). In *Drosophila*, studies of Trol in the larval lymph gland have suggested that this HSPG sequesters FGF ligands to downregulate FGF signaling within this tissue (Dragojlovic-Munther and Martinez-Agosto 2013). However, *trol* mutant phenotypes in the *Drosophila* early embryo had not previously been investigated. HSPG Sdc function was studied in late embryogenesis to examine its role in supporting cardiogenesis, and it was noted that mutants exhibit mesoderm spreading defects earlier (Knox *et al.* 2011). Here, we compare and contrast the roles of Trol and Sdc during several FGF-dependent processes in early development of the *Drosophila* embryo.

## Materials and Methods

### Fly strains and genetic crosses

*P{GAL4-twi.G}*, *w^1^* (BDSC #914) and *w*; *P{GawB}69B* (#1774) lines were used in experiments analyzing mesoderm spreading. For screening, females from “virginator” *y^1^ w/Dp(2;Y)G*, *P{hs-hid}Y* (#8846) versions of these *Gal4* stocks were crossed with males from the UAS insertion collection. Wild-type refers to *yw* or *Gal4* lines. Mutant strains were crossed with balancers containing a *lacZ* marker to identify homozygous embryos: *Sp^1^/CyO*, *P{wingless-lacZ}* (Kadam *et al.* 2009) or *Dr^Mio^/TM3*, *P{ftz-lacZ}* (#3218).

For germline clones, *trol^G011^,FRT.19A/FM7* were crossed with *P{ovoD1-18}P4.1*, *P{hsFLP}12*, *y^1^ w^1118^ sn^3^ P{neoFRT}19A/C(1)DX*, *y^1^ w^1^ f^1^* (#23880) and allowed to lay for approximately 12 hr at 25°. A 2-hr heat shock at 37° was performed on days 2, 3, and 4. Non-Bar females were then crossed with *y^1^ arm^4^ w/FM7c*, *P{ftz/lacC}YH1* males (#616) and collected embryos were analyzed. The zygotic lethality exhibited by *trol^G011^* can be rescued by a Trol duplication on the Y chromosome (#4284; data not shown). A similar protocol was used with *sdc^2639^*, *FRT42B/CyO* (Stork *et al.* 2014) and *hsFLP/Y*; *ovoD 42B/CyO* (#1929 x #4434) to generate *sdc* germline clones (maternal loss-of-function), but then crossed to males of the same genotype (*i.e*., *sdc^2639^*, *FRT42B/CyO*) to generate embryos (∼half) devoid of zygotic *sdc* (Chou and Perrimon 1996).

The *5053-Gal4* driver *w*; *P{GawB}tey5053A/TM6B*, *Tb+* (#2702) was used for ectopic expression in the CVM cells (Reim *et al.* 2012). *bHLH-gap-Venus* (Y.-K. Bae and A. Stathopoulos, unpublished data) is a transgene used as a reporter to detect CVM cells with a GFP antibody; the same enhancer has been shown previously to support expression within CVM cells (Kadam *et al.* 2012). Additional stocks, including the lines from the CSS collection (Kurusu *et al.* 2008), are listed in Supporting Information, Table S1.

UAS insertions for all genes were confirmed through expression assay. Sim-Gal4 (Xiao *et al.* 1996) or ZenKr-Gal4 (Frasch 1995), which support ectopic expression at the embryonic midline or trunk region, respectively, were used to drive expression from the insertions and *in situ* hybridization experiments confirmed ectopic expression of genes (data not shown).

### *In situ* hybridization and immunohistochemistry

Embryos were collected and aged at 25° or 18° to obtain stages of interest, and standard protocols for fixing and staining were used. Antisense RNA probes were made to detect *in vivo* gene expression using *in situ* hybridization. For antibody stainings, primary antibodies used were the following: rat anti-Twist (1:200; this study); rabbit anti-Eve (1:100; M. Frasch, University of Erlangen, Germany); mouse anti-dpERK (1:150; Sigma); rabbit anti-β-galactosidase (1:200; Molecular Probes); mouse anti-αPS2 (1:10; Developmental Studies Hybridoma Bank); and rabbit anti-GFP (1:2000; Life Technologies).

### Sample preparations and imaging

For cross-sections, stained embryos were embedded in araldite (Electron Microscopy Sciences). The 10- or 20-μm slices were sectioned using the LKB Bromma 2218 Historange Microtome and mounted in 1:1 araldite:acetone solution. For cuticle preparations, 24-hr-old embryos were dechorionated in bleach, devitillinized in 1:1 MeOH:heptane, and mounted in lactic acid. Slides were incubated at 55° overnight. All images were collected using a Zeiss Axioplan microscope.

## Results

### Ectopic expression screen identifies genes in multiple pathways affecting mesoderm development

**Figure 1:**
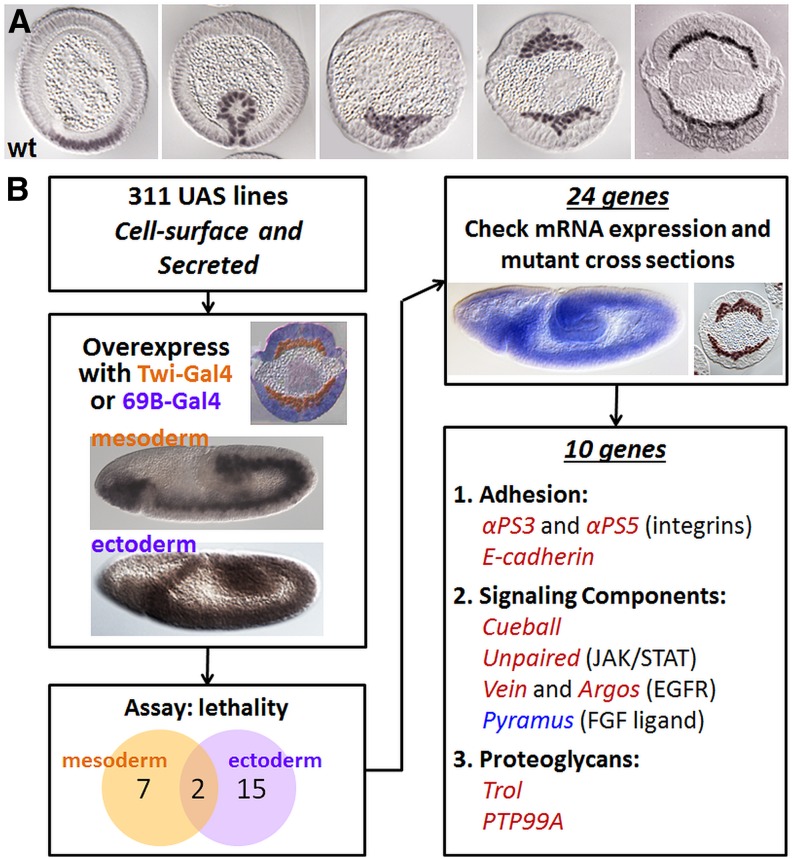
Ten genes identified by the ectopic expression screen confer mesoderm migration defects. (A) Cross-section of *Drosophila* embryos stained with Twist antibody to mark mesoderm cells during development, ventral side down (shown here and in other figures unless otherwise noted). In stages 5–10, the mesoderm invaginates to form the ventral furrow, which subsequently collapses onto the ectoderm. Dorsal migration follows and the process is complete after intercalation helps to specify a monolayer. (B) Workflow of ectopic expression screen. Cell-surface and secreted (CSS) proteins were overexpressed in the mesoderm using Twi-Gal4 and in the ectoderm using 69B-Gal4. Candidates were narrowed to 10 genes based on their RNA expression and mutant phenotypes: *pyramus* (blue), which previously had been characterized for its role in mesoderm migration (McMahon *et al.* 2010), and nine novel genes (red). Genes comprise three different classes: adhesion, signaling components, and proteoglycans. Here, and in all following figures, lateral views of whole-mount embryos are positioned with anterior facing left and ventral side facing down. Cross-section depicting Gal4 lines taken from Kadam *et al.* (2009).

Presumptive mesoderm cells are initially specified prior to gastrulation in ventral regions of the embryo (Reeves and Stathopoulos 2009; Solnica-Krezel and Sepich 2012). These ventral cells undergo shape changes that cause a furrow to form that comprises the presumptive mesodermal domain. Apical constriction of cells drives their invagination, during which time a tube is formed on the inside of the embryo. Cells undergo epithelial-mesenchymal transition (EMT) and, subsequently, the invaginated tube collapses on the inner surface of ectodermal cells. These presumptive mesoderm cells then migrate in the dorsal direction, and at the end of the process they undergo small movements (intercalations) toward the ectoderm to establish a single layer of mesoderm cells on the inside of the embryo (Figure 1A).

To elucidate potential signaling pathways and adhesion molecules that influence mesoderm migration, we conducted a screen of a library comprising 311 insertions at the presumed 5′ end of genes encoding cell surface or secreted (CSS) factors (Figure 1B). These lines were previously selected to help with identification of extracellular-acting signaling molecules and used in a screen of neuronal targeting (Kurusu *et al.* 2008). Using these fly stocks in the current study, we aimed to identify novel regulators of mesoderm spreading during gastrulation. To this end, genes were overexpressed using Gal4 drivers that support expression in the mesoderm (Twi-Gal4) (Kusch and Reuter 1999) or ectoderm substratum (68B-Gal4) (Brand and Perrimon 1993) (Figure 1B). Twenty-four insertions were identified that caused lethality upon ectopic expression in the mesoderm and/or ectoderm (Table 1).

**Table 1:** Twenty-four genes found to confer embryonic lethality when ectopically expressed in the ectoderm or mesoderm

Lethality	Gene ID	Name	Abbreviation	UAS Line	Endogenous Expression, Stages 5–10
Twi-Gal4	CG8084	Anachronism	Ana	GS 9498	Mesoderm
	CG1106	Gelsolin	Gel	GS 10156	Mesoderm, gut
	CG8434	Lambik	Lbk	GS 17119	Ectoderm
	CG7476	Methuselah-like 7	Mthl7	GS 21256	Weak mesoderm*^c^*
	CG9342	Microsomal triacylglycerol transfer protein	Mtp	XP d07488	Mesoderm, yolk
	CG2005	*^a^*Protein tyrosine phosphatase 99A	Ptp99A	EY 7423	Mesoderm
	CG8095	*^a^*αPS3 integrin/Scab	Scb	EP 2591	Mesoderm, gut
69B-Gal4	CG5372	*^a^*αPS5 integrin	ItgαPS5	GS 12413	Mesoderm
	CG4531	*^a^*Argos	Aos	GS 12984	Mesoderm
	CG12086	*^a^*Cueball	Cue	EY 1263	Mesoderm, gut
	CG15013	Dusky-like	Dyl	GS 20894	Weak mesoderm
	CG3722	*^a^*E-cadherin/Shotgun	Shg	XP d01606	Ectoderm
	CG32356	Ecdysone-inducible gene E1	ImpE1	GS 11510	Weak mesoderm
	CG32464	l(3)82Fd/Mustard	Mtd	GS 16948	n/d*^d^*
	CG13194	*^a^*Pyramus	Pyr	GS 22603	Ectoderm
	CG5661	Semaphorin-5c	Sema-5c	EY 1704	Mesoderm stripes, gut
	CG33950	*^a^*Terribly reduced optic lobes	Trol	GE 10067	Mesoderm, ectoderm
	CG6890	Toll-8/Tollo	Tollo	XP d01565	Ectoderm stripes
	CG5528	Toll-9	Toll-9	GS 51	Weak mesoderm
	CG9138	Uninflatable	Uif	GS 11655	Ectoderm stripes
	CG34056	galactosyltransferase		GS 11028	Weak mesoderm
	CG9550	sulfotransferase		GS 18034	Weak mesoderm*^b^*
Twi- and 69B-Gal4
	CG5993	*^a^*Unpaired/Outstretched	Upd/Os	G17133	Ectoderm stripes
	CG10491	*^a^*Vein	Vn	GS 12044	Ectoderm

a Total of 10 genes were identified that had relevant (*i.e.*, mesoderm or ectoderm) endogenous RNA expression and had mutant mesoderm defects: nine novel genes plus the FGF ligand Pyramus, which has previously been shown to function in mesoderm migration (Kadam *et al.* 2009).

b Berkeley *Drosophila* Genome Project (BDGP) reports zero expression of *CG9550* at embryonic stages 5–10.

c No developmental timecourse of expression data available for *mthl7* at BDGP.

d Low to moderate expression for *mtd* is reported at embryonic stages 5–10 at BDGP.

Next, we screened these candidates to determine if lethality was caused by defects in mesoderm migration. Lethality could also relate, instead, to a dominant negative effect where ectopic expression of genes, even if not normally acting to affect mesoderm migration, may compete with normal processes. Therefore, candidate genes were selected that were expressed within embryonic domains that could impact mesoderm migration, meaning genes were expressed (i) during stages 5–10 encompassing invagination of the mesoderm through monolayer formation and (ii) within the migrating mesoderm and/or in proximity to the mesoderm within the ectoderm. We then examined embryo cross-sections for spreading defects in loss-of-function mutant backgrounds for this set of genes (Figure 1B). Single null mutants were examined if available; if not, then deficiency chromosomes deleting the gene in question (along with many others) were assayed. We reasoned that genes normally acting to support the mesoderm spreading processes would exhibit mutant phenotypes.

**Figure 2:**
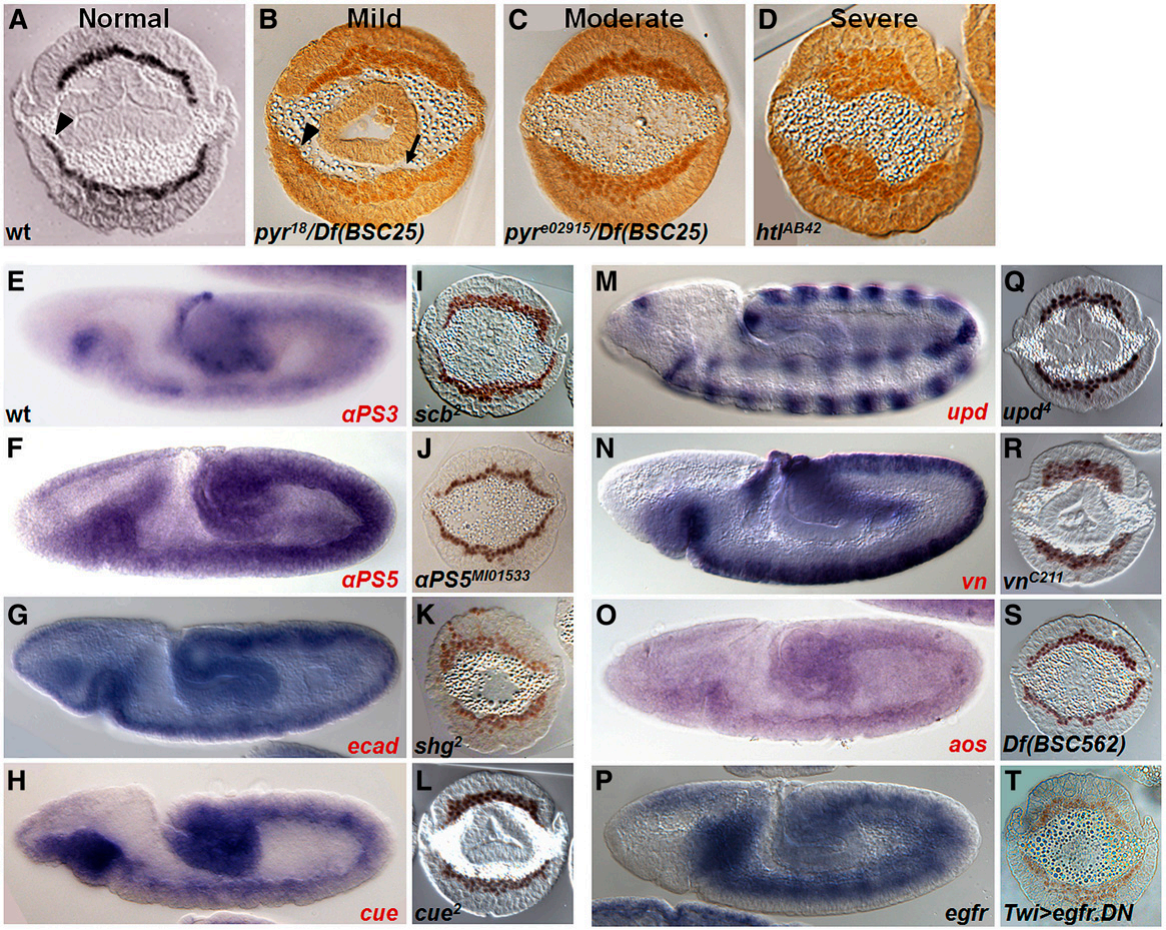
Endogenous expression and mutant phenotypes of adhesion molecules and signaling components isolated from the screen. Cross-sectioned embryos are of stage 9–10 when mesoderm cells are at the end of their migration. (A–D) A comparison of wild-type with mild, moderate, and severe mesoderm spreading phenotypes. (A) Wild-type embryos have a monolayer of mesoderm cells. The arrowhead marks where the mesoderm cells have reached the dorsal region of the embryo, where cells receive additional differentiation signals. (B) *pyr^18^/Df BSC25 trans*-heterozygous mutant embryos have a mild phenotype marked by regions where mesoderm cells are multilayered (arrow). However, some cells intercalate into a single layer (arrowhead). (C) *pyr^e02915^/Df BSC25* embryos have a moderate phenotype where the mesoderm is uniformly multilayered. *Df BSC25* is a deficiency that encompasses both Pyr and Ths, FGF ligands for the FGFR Htl. (D) *htl^AB42^* mutants have severe defects such that the mesoderm forms lumps of cells. (E–T) Preliminary expression and mutant analysis of genes isolated in the screen. RNA expression patterns in wild-type embryos of stage 8–9 (lateral views: E–H, M–P) and cross-section of zygotic mutant embryos showing α-Twi expression to mark mesoderm (cross-sections: I–L, Q–T). Single mutants were assayed if available (I, J, K, L, Q, R) for genes isolated from the ectopic expression screen; otherwise, data for deficiencies are shown (*aos*: S). For assay of *egfr*, the dominant negative (DN) form of *egfr* was overexpressed using the Twi-Gal4 driver (T). *In situ* hybridization was performed using riboprobes specified for the indicated genes. Genes in red denote those isolated from this screen.

These phenotypes were classed into three different levels of severity (Figure 2, A–D). Mild indicates only a few cells did not intercalate, creating an uneven layer. Mesoderm defects of moderate phenotype present multilayered cells, which nevertheless evenly spread on the ectoderm but fail to form a monolayer. Severe phenotypes include uneven spreading of cells on the ectoderm (*i.e.*, not centered at the midline) as well as multilayered/clumping of cells, as in the case with *htl* mutants, which exhibit defects in mesoderm collapse, spreading, and intercalation (McMahon *et al.* 2008). Also, we have observed that *htl* phenotypes are variable, ranging from mild to severe (Table 2, see *htl*). To quantify phenotypes that could be variable, at least 7 and as many as 23 embryos were examined for mutants assayed. Furthermore, a score was calculated based on frequency of phenotypes observed (Table 2). In a recent study, cadherin mutants were found to exhibit nonmonolayer phenotypes, but a role for these molecules in supporting mesoderm formation was dismissed because germ layers were specified (Schafer *et al.* 2014). Because the goal of our screen was to uncover signals guiding proper mesoderm migration, lack of a monolayer is relevant and indicates defects in effective mesoderm migration. For this reason, we considered mutant phenotypes that span the range of mild to severe.

**Table 2:** Quantification of mesoderm spreading phenotypes in mutant embryos

Mutant	Normal = Score 1	Mild = 2	Moderate = 3	Severe = 4	Total *n*	Average Score	Representative
*yw* (wild-type)	*n* = 8	1	0	0	9	0.9	Normal
*htl^AB42^*	1	3	3	6	13	**3.1**	**Moderate/severe**
*pyr^e02915^/Df BSC25*	2	2	3	4	11	2.8	Moderate
*pyr^18^/Df BSC25*	2	5	3	0	10	2.1	Mild
*scab^2^* (αPS3)	1	10	4	2	17	2.4	Mild
*αPS5^MI01533^*	1	3	2	1	7	2.4	Mild
*shg^2^* (Ecad)	0	4	4	6	14	**3.1**	**Moderate/severe**
*cue^2^/Df*	2	4	2	0	8	1.8	Mild
*upd^4^*	0	3	8	0	11	2.7	Moderate
*vn^c211^*	0	2	6	0	8	2.8	Moderate
*aos (Df BSC562)*	1	6	5	2	14	2.6	Moderate
*Twi > egfr.DN*	2	1	6	0	9	2.4	Mild
*Df BSC354 (CG9550)*	0	5	3	2	10	2.7	Moderate
*Df Exel6086 (CG34056)*	0	4	4	0	8	2.5	Moderate
*ptp99a^1^*	3	3	1	0	7	1.7	Mild
*trol^G011^ m-z-*	2	5	6	9	22	**3.0**	**Moderate/severe**
*sdc^2639^ m-z*- and *m-z+*	9	8	6	0	23	1.9	Mild

Stage 9–10 embryos were stained with Twist antibody to mark the mesoderm and cross-sectioned to determine any mesoderm spreading defects (Figure 2). Embryos mutant for genes identified from the screen, in addition to embryos mutant for the FGF pathway as controls, were tallied. A weight was given for each phenotypic class and averaged. Only three genes, *htl*, *shg*, and *trol*, had a score of 3 or more; therefore, their mesoderm migration phenotypes were classified as moderate/severe. “Moderate” phenotypes had a score of 2.5–2.9 and “mild” phenotypes had a score of 1.5–2.4. A score less than 1.4 was classified as “normal” mesoderm spreading. *Df BSC25* uncovers both genes *pyr* and *ths*, encoding ligands for the FGFR Htl.

Screening in this manner identified 10 genes of interest that include the FGF ligand Pyramus (Table 1, footnote "a"). These 10 genes had both relevant expression patterns (*i.e.*, endogenous mesoderm and/or ectoderm expression) and mutant phenotypes relating to mesoderm migration. Spreading defects for these 10 genes as well as a number of controls, genes previously implicated in mesoderm migration (*i.e.*, *htl^AB42^*, *pyr^e02915^/BSC25*, and *pyr^18^/BSC25*), were scored and quantified into the different levels of severity: normal, mild, moderate, or severe (Figure 2, A–D, Table 2).

### Classes of signaling components and adhesion molecules known to be regulators of mesoderm migration during gastrulation were identified

Genes encoding one FGF ligand, two integrins, and one cadherin were identified by the screen; these genes were expected and support previous roles in facilitating mesoderm migration during gastrulation (McMahon *et al.* 2010; Clark *et al.* 2011). An insertion upstream of the FGF ligand Pyr (GS22603) resulted in embryonic lethality upon ectopic expression with the 69B-Gal4 driver (data not shown). A previous study characterized the role of the FGF ligand Pyr in supporting monolayer formation (Kadam *et al.* 2009).

In addition, prior studies also identified a role for the β-PS integrin Mys in this process, demonstrating that it is required solely for monolayer formation at the end of the migration following spreading of cells on the ectoderm (McMahon *et al.* 2010). In the current screen, two alpha integrins, α-PS3 (Scab; EP2591) and α-PS5 (GS12413), were identified that may act with the β-PS integrin Mys. Integrins function in tetramers with the binding of two α- and two β-integrins (Bulgakova *et al.* 2012). Ectopic expression of α-PS3, using the mesoderm driver, or αPS5, using the ectoderm driver, was lethal; expression of each in the alternate tissue was not (Table 1). Both genes, α-PS3 and α-PS5, are expressed in the mesoderm, and single mutants affecting each of these integrins show mild spreading defects (Figure 2, E, F, I, J) supporting the view that both act to facilitate mesoderm spreading. The tissue-specific effects observed for ectopic expression suggest that the balance of integrin subunits is important. For instance, it is possible that multiple integrins, including Mys, α-PS3, and α-PS5, as well as others, act collectively or redundantly to support mesoderm migration during gastrulation through effects on regulation of adhesion and/or signaling state. *Drosophila* contains three additional α-integrins, all of which are present during early mesoderm development (Figure S1, A–C).

Finally, E-cadherin (Ecad, *Drosophila* Shotgun) was isolated. Cadherins play pivotal roles in controlling adhesion and epithelial-to-mesenchymal transition (EMT) (Oda and Tsukita 1999). Ecad is expressed in the ectoderm at gastrulation when mesoderm migration occurs (Oda *et al.* 1998), and mutants exhibit a severe mesodermal phenotype (Figure 2, G and K and Table 2).

### Newly identified regulators of mesoderm migration include signaling components and adhesion molecules

Because these genes had already been linked to control of cell movements during gastrulation, we focused our analyses on other genes that might provide novel insights into this process.

Only two genes induced embryonic lethality when overexpressed in either the mesoderm or the ectoderm. Both of these genes encode secreted factors and ligands influencing signaling pathways: Unpaired (Upd; G17133) (Figure S1, D and E) regulates the JAK/STAT pathway and Vein (Vn; GS12044) regulates EGFR signaling. Although previous studies have focused on *upd* during heart diversification (Johnson *et al.* 2011), a role in the early mesoderm at gastrulation had not been identified. Upd is expressed in ectodermal stripes and mutant embryos result in a moderate multilayer phenotype (Figure 2, M and Q). Modulation of other JAK/STAT signaling components had mild to moderate effects on mesoderm migration (Figure S1, F–H). However, although the Upd receptor Domeless (Dome) is expressed in the mesoderm, *dome* mutants do not result in any spreading defects (Figure S1, I–L). It is possible that Dome and JAK/STAT signaling are required later in the mesoderm after migration is complete.

The second secreted molecule that resulted in embryonic lethality when ectopically expressed in either the mesoderm or the ectoderm was Vein, an epidermal growth factor receptor (EGFR) ligand (Figure S1, M and N). Normally, *vn* is expressed in the ectoderm, and *vn* mutants exhibit a moderate mesoderm phenotype (Figure 2, N and R). Another EGF pathway component, Argos (Aos), was also identified in the screen. Aos is expressed in the mesoderm and the deficiency lacking *aos* also presented a moderate mesoderm spreading phenotype (Figure 2, O and S). Because two components of the EGFR pathway were identified in the screen, we also examined the phenotype associated with the receptor itself (Shilo 2005). EGFR is upregulated in the mesoderm when spreading is complete, and expressing its dominant negative form in the mesoderm resulted in a mild phenotype (Figure 2, P and T). However, *egfr* mutants and ectopic expression of the EGFR dominant negative in the ectoderm had little to no effect on the mesoderm even though EGFR is present in the ectoderm at earlier stages (Figure S2, O–T).

It is possible that the JAK/STAT and EGFR signaling pathways are active in the mesoderm during migration. Future studies may distinguish direct from indirect roles; for instance, these pathways may regulate gene expression and/or protein distributions of other genes within the ectoderm required to instruct mesoderm migration.

We identified an insertion (EY1263) near the *cueball* (*cue*) gene, which encodes a membrane-bound protein that is EGF-like and contains LDLR repeats. It is expressed in the mesoderm, and embryos lacking *cue* exhibit a mild phenotype (Figure 2, H and L). It is possible that Cue supports localization of secreted or membrane proteins, because previous studies suggest it impacts vesicle trafficking (Hirst and Carmichael 2011).

Our screen also isolated additional genes that were either previously uncharacterized and/or had unknown functions (Table 1 and Figure S2). Two are predicted enzymes, a sulfotransferase *CG9550* (GS18034) and a galactosyltransferase *CG34056* (GS11028). Analyses of these two genes show weak mesoderm expression and spreading defects when analyzed in the context of deficiency chromosomes (Figure S2, A and B). However, more than 20 genes were uncovered by these large deletions; therefore, it is unclear whether these phenotypes directly relate to the genes in question. However, expression of RNAi targeting these genes and/or ectopic expression results in moderate defects providing additional support for a role for these genes in supporting mesoderm migration (Figure S1, U–Y). These enzymes could potentially function in the synthesis and/or modification of proteoglycans, which were also found in the screen (see below). In addition, two genes from the Toll family of receptors, which can spatially influence heterophillic cell–cell interactions (Pare *et al.* 2014), were also identified. However, these genes and the others identified require further characterization to determine whether they impact mesoderm spreading directly (see Table 1 and Figure S2).

### Comparing proteoglycans in the *Drosophila* embryo

**Figure 3:**
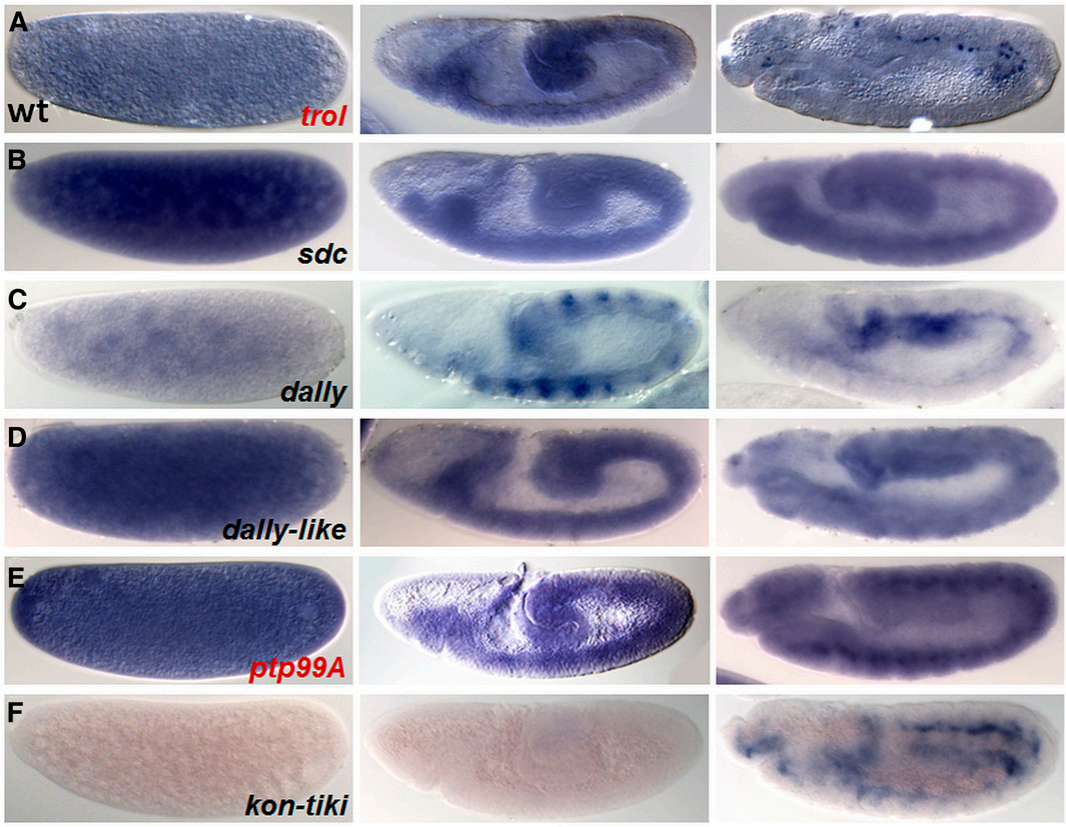
Embryonic expression of *Drosophila* proteoglycans is dynamic. Wild-type expression patterns for HSPGs detected using *in situ* hybridization and specific riboprobes for: (A) *trol*; (B) *sdc*; (C) *dally*; (D) *dally-like* and putative CSPGs: (E) *ptp99a*; and (F) *kon*. Lateral views of embryos staged at pre-cellularization (left column), gastrulation (stage 8; middle), and in germ-band elongated embryos (stage 12; right).

Proteoglycans have a variety of activities that directly and indirectly modulate signaling, including the FGF pathway; however, their role in mesoderm migration has not been fully investigated. The *Drosophila* genome contains four HSPGs: Trol, Sdc, Dally, and Dlp (Lin 2004). Trol was identified in our screen, and Sdc was previously reported to play a role in mesoderm development in the embryo (Knox *et al.* 2011). In addition, there are two predicted chondroitin sulfate proteoglycans (CSPGs) based on sequence homology: PTP99A and Kon-tiki (Kon) (Song *et al.* 2012). Our screen also isolated Ptp99A and, although it is unclear if Ptp99a is a true CSPG (see *Discussion*), we proceeded to investigate both these families of proteoglycans more closely for their embryonic expression. All genes, except *kon*, are maternally deposited and are expressed during mesoderm migration (Figure 3). In addition, Trol and Kon are expressed in what appears to be the caudal visceral mesoderm (CVM), another group of FGF-dependent migrating cells that undergo migration at later stages after gastrulation (Kadam *et al.* 2012).

**Figure 4:**
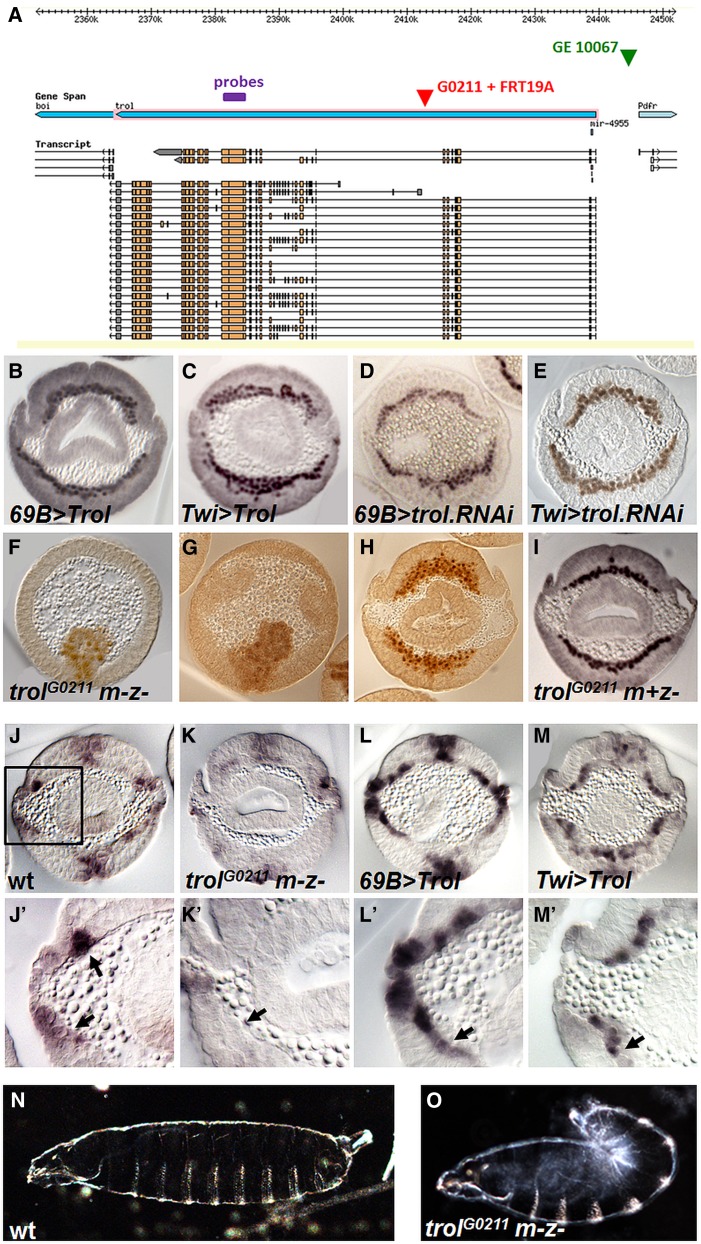
*trol* germline clones exhibit defects in mesoderm migration similar to FGF mutants. (A) Image of *trol* locus obtained from Flybase GBrowse depicting location of the reagents used in this study: GE10067 is a UAS insertion and G0211 is a *lacZ* insertion that was recombined with FRT 19A to support generation of germline clones. (B–E) Mesoderm migration phenotypes in embryo cross-sections (stage 9–10) detected using α-Twi antibody resulting from *trol* ectopic expression and tissue-specific downregulation . Ectopic expression of *trol* in the ectoderm (B) or mesoderm (C) results in mesoderm spreading defects. Downregulation of *trol* levels by tissue-specific RNAi in the ectoderm (D) or mesoderm (E) also yields spreading defects. (F-I) *trol* maternal plus zygotic mutant phenotypes in a time-course of embryo cross-sections of stage 6 (F), stage 7 (G), and stage 9–10 (H) detected using α-Twi antibody. While invagination is normal (F), defects in EMT are observed because tube collapse is nonsymmetrical (G) and the mesoderm remains multilayered even at stage 10 (H). Zygotic mutants have a mild phenotype (I), thus maternal *trol* contributes to the spreading defects observed. (J–M) α-dpERK stainings in cross-sections of stage 10 embryos also showing magnified views (J′–M′). In wild-type embryos (J), FGF-dependent dpERK is found in only two or three of the dorsal most mesoderm cells (J′, arrows). This staining is absent in *trol* germline clones (K, arrow). In embryos where *trol* is overexpressed in the ectoderm with the 69B-Gal4 driver, dpERK staining has expanded to 5 or more cells (L, arrow). Embryo ectopically expressing *trol* in the mesoderm with Twi-Gal4 shows ectopic dpERK throughout the mesoderm (M, arrow). (N, O) Cuticles associated with wild-type (N) or *trol* germline clones that display a “tail-up” phenotype (O).

The *trol* locus spans ∼75 kB and includes as many as 58 exons encoding 22 unique polypeptides (Figure 4A). Ectopic expression of UAS-Trol^GE10067^ or *trol* RNAi (Grigorian *et al.* 2013) constructs in either the ectoderm or the mesoderm results in mild to moderate spreading defects (Figure 4, B–E). Germline clones devoid of both maternal and zygotic (m-z-) *trol* transcripts exhibit mesoderm tube collapse defects (compare Figure 1A with Figure 4, F and G) that result in a severe mesoderm phenotype (Figure 4H). Furthermore, we show that maternal Trol contribution is sufficient to rescue the collapse defect and partially rescues the spreading phenotype to mild (Figure 4I). These results suggest that maternal Trol contribution supports early mesoderm migration, namely tube collapse, whereas zygotic Trol influences monolayer formation (and likely additional subsequent functions). It is possible that localized expression and/or increased levels of Trol, supported by zygotic transcription, is necessary for proper monolayer formation (see *Discussion*).

Importantly, the phenotype in *trol* germline clones is similar to that found in embryos lacking FGF signaling (*e.g.* McMahon *et al.* 2008). We therefore investigated whether FGFR receptor activation was possible in the absence of Trol by assaying for dpERK expression in the mesoderm. dpERK is a measure of RTK intracellular signaling activation. At the end of gastrulation, dpERK staining is present within a subset of mesoderm cells that have migrated to the dorsal-most position (Figure 4, J and J′, arrows) as well as in patches within the ectoderm; mesodermal and ectodermal dpERK staining has been demonstrated to relate to FGFR *vs.* EGFR RTK activation, respectively (Gabay *et al.* 1997b). We found that dpERK is absent from the mesoderm in embryos from *trol* germline clones (Figure 4K). Furthermore, when *trol* is overexpressed in the ectoderm or mesoderm, dpERK is expanded or ectopically expressed, respectively (Figure 4, L and M). Trol may also support other signaling pathways because embryos lacking *trol* had an overall reduction of EGFR-dependent dpERK in ectodermal cells (Figure 4K). In addition, *trol* germline clones exhibit a “tail-up” phenotype, suggesting an additional role related to TGF-β signaling, possibly at a later stage (Figure 4, N and O, see *Discussion*) (Ferguson and Anderson 1992).

### Trol and Sdc have different roles in embryonic development

**Figure 5:**
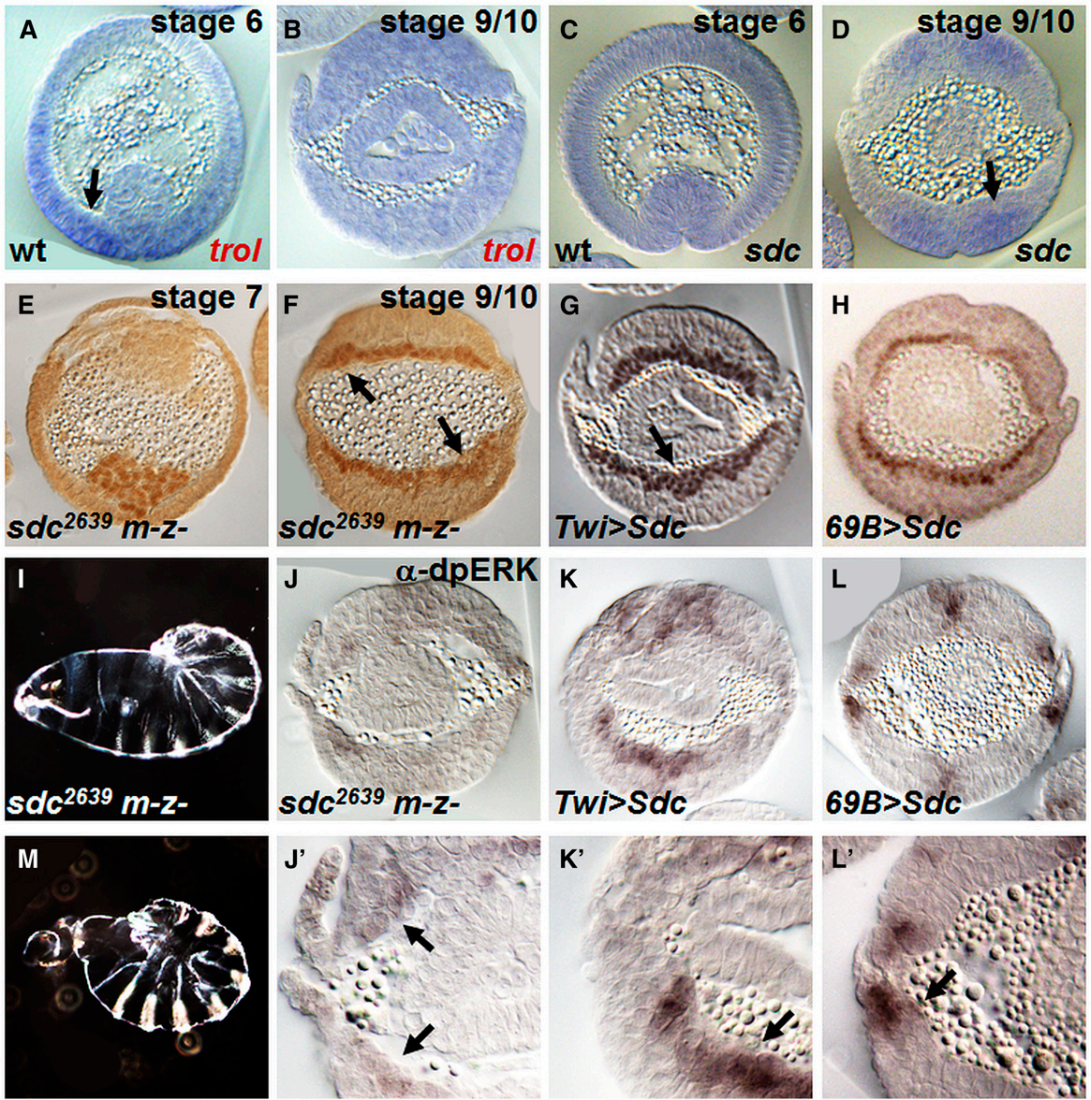
*sdc* mutant embryos exhibit mild defects in mesoderm migration. (A–D) *In situ* hybridization of (A, B) *trol* and (C, D) *sdc* in wild-type embryos. At stage 6, *trol* is upregulated in the ventral-most ectoderm cells surrounding the invaginated furrow (A, arrow). In contrast, *sdc* is localized to the same position but at a later stage, when mesoderm cells intercalate to form a monolayer (D, arrow). (E) *sdc* germline clones have normal mesoderm collapse (*i.e.*, symmetrical). (F) *sdc* germline clones have mild spreading defects as mesoderm cells form a nonmonolayer (arrows). (G) Ectopically expressing *sdc* in the mesoderm results in a multilayered mesoderm (arrow). (H) Overexpressing *sdc* in the ectoderm has little effect as mesoderm spreading appears normal (*i.e.*, monolayer). (I, M) *sdc* germline clones exhibit a range of cuticular phenotypes that range from "tail-up" to twisted/loss-of-head. (J, K) The α-dpERK staining is detected in cross-sections of embryos from *sdc* germline clones within dorsal-most mesoderm cells (J; magnified view: J′, arrows), possibly at a reduced level compared with wild-type (see Figure 4J). Ectopic expression of *sdc* within the mesoderm results in ectopic dpERK throughout the tissue (K; K′, arrow), whereas overexpression of Sdc in the ectoderm has little effect (L; L′, arrow).

Because both Trol (Figure 4) and Sdc (Knox *et al.* 2011) mutants exhibit phenotypes that affect the mesoderm of early embryos, we investigated their expression patterns during the stages of early mesoderm development to provide more specific insights into these genes' functions. Both genes are maternally expressed and present ubiquitously at low levels; however, at two stages, localized expression was detected. Once the furrow is formed, *trol* is upregulated in the ventral-most cells where the mesoderm will collapse onto the ectoderm; in contrast, Sdc at this stage remains ubiquitously diffuse (compare Figure 5A arrow and Figure 5C). Conversely, *sdc* becomes localized to the ectoderm later, when the mesoderm intercalates to form a single layer of cells (Figure 5D arrow); in contrast, *trol* at this later stage is no longer spatially upregulated and instead is present uniformly at low levels (Figure 5B). The dynamics of *sdc* expression suggest that Sdc, like zygotic Trol, may be required only for monolayer formation at later stages.

In accordance with the *sdc* expression pattern, *sdc^2639^* germline clones (Stork *et al.* 2014) exhibit normal collapse during early mesoderm migration (compare Figure 1A with Figure 5E). However, at later stages, these embryos have mild spreading defects often seen when cells are unable to intercalate to form a monolayer (Figure 5F) (Knox *et al.* 2011). Nevertheless, FGF-dependent dpERK staining within the mesoderm is present in *sdc* germline clones (Figure 5J). Ectopic expression of *sdc* in the mesoderm results in a moderate phenotype and leads to dpERK presence throughout the mesoderm (Figure 5, G and K). In contrast, increasing *sdc* in the ectoderm where it is already expressed has little to no effect on mesoderm spreading or on dpERK activation (Figure 5, H and L). Unlike with *trol*, EGFR-dependent dpERK expression in the ectoderm does not appear to change in either *sdc* germline clones or overexpression of Sdc (Figure 5, J–L). However, *sdc* germline clones do have severe cuticle phenotypes similar to *trol* mutants (Figure 4, I and M), indicative of TGF-β signaling defects.

Furthermore, ectopic expression of other HSPGs Dally and Dally-like display mild or no mesoderm spreading defects (Figure S3, A–H). Whereas overexpression of Ptp99a in the mesoderm resulted in a moderate spreading phenotype, removing *ptp99a* in the embryo had little effect (Figure S3, I–K). Kon was not examined because this gene is not expressed until later embryonic stages and thus does not regulate mesoderm migration (Figure 3F). Therefore, the roles of Trol and Sdc in supporting mesoderm migration are specific and not shared by other HSPGs. In addition, both FGF and EGFR signaling, as assayed by dpERK activation, appear to be affected by Trol and Sdc in different ways.

### Trol and Sdc in other FGF-dependent processes

**Figure 6:**
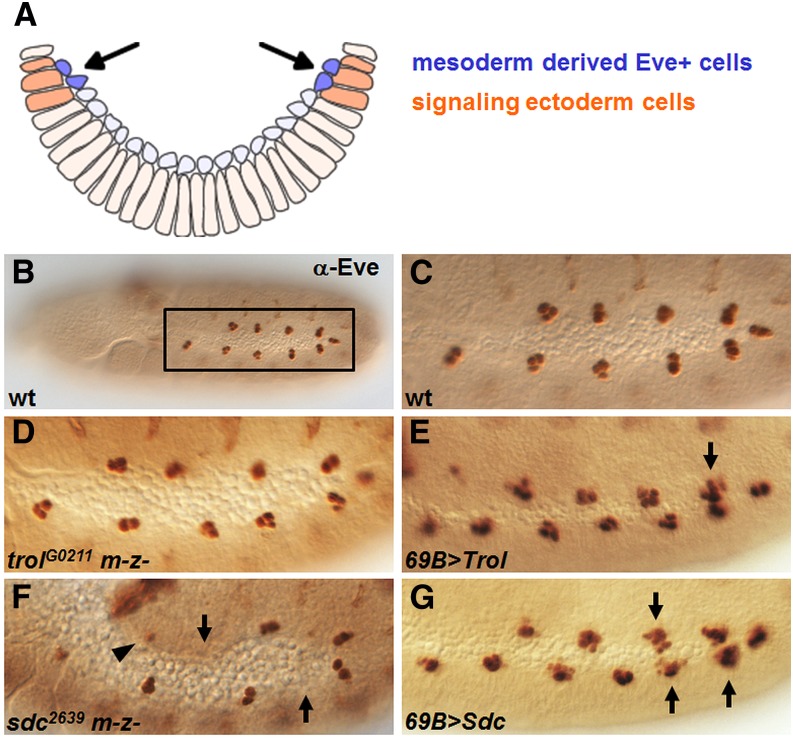
Embryos obtained from *sdc*, but not *trol*, mutant germline clones exhibit defects in Eve specification. (A) Schematic cross-section of the ventral half of an embryo at stage 11 during Eve specification. Mesoderm cells that reach the dorsal regions of the embryo (arrows) are able to receive signals, including FGF, from the ectoderm (dark orange) and undergo cell differentiation (dark blue). (B–G) Dorsal somatic mesoderm cell differentiation at stage 11 embryos is marked by Eve expression. (B) Wild-type whole embryo stained using anti-Eve antibody includes box showing region of magnification in subsequent panels. (C) Wild-type embryos have 10 clusters of three Eve^+^ cells. (D) *trol* germline clones show a normal number of Eve^+^ cells. (E) Embryos overexpressing *trol* in the ectoderm occasionally have clusters with four Eve^+^ cells, as indicated by the arrow. (F) *sdc* germline clones are missing clusters (arrows) and/or have reduced number of Eve^+^ cells within a cluster (arrowhead). (G) Embryos with *sdc* overexpressed in the ectoderm have multiple clusters with five or more Eve^+^ cells (arrows).

Pericardial and dorsal somatic muscle cells derived from the dorsal mesoderm are known to express Even-skipped (Eve) (Frasch 1999) and require proper migration of the mesoderm at an earlier stage prior to their specification. Once mesoderm cells migrate to dorsal regions of the ectoderm (Figure 2A arrowhead; Figure 6A arrow), they are induced by signals originating from the ectoderm to express Eve within 10 clusters of three cells each, spanning the trunk of the embryo (Figure 6, B and C). These differentiation cues include FGF, Wg, and Dpp—all of which have the ability to cooperate with HSPGs in the context of receptor activation (Lin 2004). In both *trol* and *sdc* mutant embryos, mesoderm cells reach the dorsal ectoderm as a result of their migration despite their nonmonolayered spreading (Figure 4H and Figure 5F). We examined if Trol is required for the subsequent patterning of dorsal somatic lineages, but no measurable defect in Eve-specification was observed in embryos derived from *trol* germline clones (Figure 6D). Previous studies, however, have shown that *sdc* zygotic mutants, in contrast, do exhibit defects in Eve induction and linked this to effects on FGF signaling through genetic interaction assay (Knox *et al.* 2011). Likewise, we found that in embryos obtained from *sdc* germline clone, a significant reduction of Eve^+^ cells was observed (Figure 6F). These results reinforce the view that Sdc is required to support the role of FGF in differentiation of dorsal somatic mesoderm lineages. Overexpression of either Trol or Sdc in the ectoderm results in increased Eve^+^ cell number. However, overexpressing Sdc had a stronger phenotype than Trol with multiple clusters containing five or more cells (compare Figure 6E with Figure 6G). Although Trol is not required to support differentiation of dorsal somatic lineages, it can potentially substitute for Sdc when ectopically expressed (compare Figure 6D with Figure 6E).

**Figure 7:**
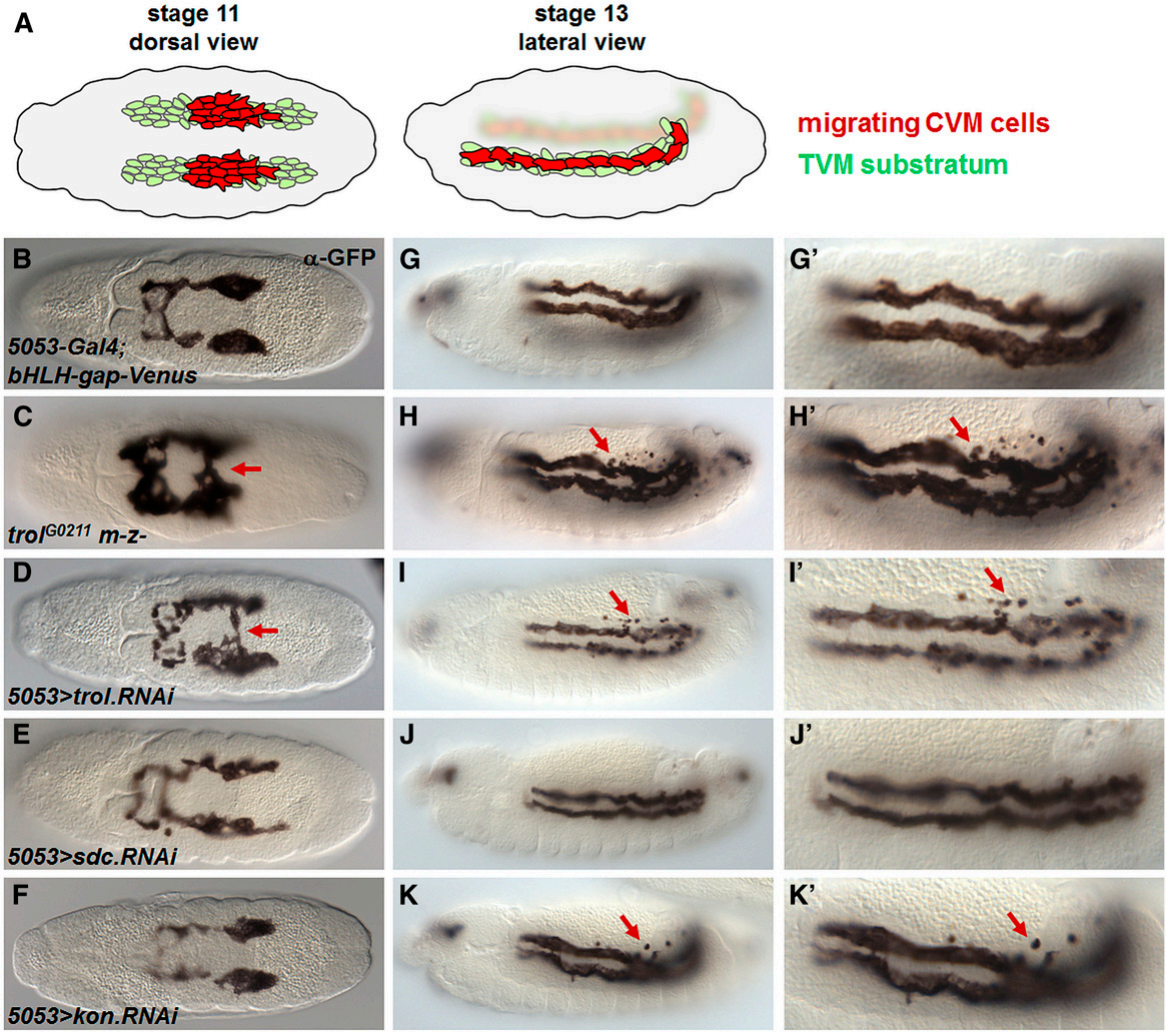
Embryos with reduced *trol* but not *sdc* levels exhibit defects in CVM migration. (A) Schematic depicting CVM migration. Red cells are the two migrating CVM clusters that express the FGFR Htl FGFR as well as the HSPG Trol (Figure 3A, right). The TVM substratum is shown in green, and this tissue expresses both FGF ligands Pyr and Ths. (A, left and B–F) Dorsal view of stage 11 embryos. (A, right and G–K) Lateral view of stage 13 embryos. (B–K) Embryos containing the CVM-specific driver 5053-Gal4 and CVM marker transgene bHLH-gap-Venus. Anti-GFP staining marks the CVM in control embryos (B, G), *trol* germline clones (C, H), and in embryos expressing RNAi hairpin constructs directed against *trol* (D, I), *sdc* (E, J), and *kon* (F, K) in CVM cells. (G′–K′) Magnified view of CVM cells to show ectopic cell death. Arrows point to merging phenotype (C, D) or ectopic cell death (H, I, K). Schematic in (A) reprinted with permission from Bae *et al.* (2012).

FGF signaling is also known to function during development of longitudinal muscle fibers (Kadam *et al.* 2012). Of the proteoglycans examined by expression analysis, we found Trol and Kon are present in a migrating population of cells originating from the CVM (Figure 3, A and F). At stages 11–13, the CVM forms two clusters of cells that migrate on the trunk visceral mesoderm (TVM) substratum. Similar to the arrangement in mesoderm migration, migrating CVM cells express the FGFR Htl, whereas the TVM substratum expresses the FGF ligands Pyr and Ths (Figure 7A) (Kadam *et al.* 2012). Both *trol* germline clones and *trol* RNAi in the CVM cells resulted in a migration defect in which cells from each of the two migrating collectives merge at the midline (compare Figure 7B with Figure 7, C and D arrows), similar to the phenotype caused by removing FGF signaling (Kadam *et al.* 2012). These *trol* mutants, along with *kon* RNAi, also exhibited increased apoptosis of CVM cells, as indicated by the punctate spots at the posterior of the embryo (compare Figure 7G with Figure 7, H, I, and K arrows). Whether this is due to a role for Trol in supporting cell survival and/or relates to mis-migration is unclear because either could account for the phenotype. Finally, introducing *sdc* RNAi into CVM cells had no effect (Figure 7, E and J), further supporting the view that Trol and Kon, but not Sdc, are required in the migrating CVM.

## Discussion

Previously, a limited number of extracellular effectors were shown to be important for mesoderm migration during gastrulation, including the FGF receptor Htl, its two FGF ligands (Pyr and Ths), and the β-PS integrin Mys (Bae *et al.* 2012). In our screen of cell surface and secreted proteins, we identified 9 additional effectors, based on mutant phenotype, as well as highlighted 14 other genes that may also play a role in supporting mesoderm migration. Some results were expected and others provide novel insight into this process. Several α-integrin genes were isolated, some or all of which may bind to known player β-integrin Mys to form tetramers. This result suggests that cell adhesion has a role in mesoderm development. Our screen also detected E-cadherin, which regulates adhesion between cells. Although other studies have suggested E-cadherin is necessary for EMT at the onset of mesoderm migration (Oda and Tsukita 1999) or for differentiation of dorsal somatic lineages rather than for supporting the subsequent process of mesoderm migration (Schafer *et al.* 2014), our results suggest E-cadherin does impacts the mesoderm spreading process as mutants display a moderate to severe phenotype that is similar to FGF mutants. Recent studies have also shown that cadherins may influence the cell’s ability to support cell signaling through modification of adhesion states (Cai *et al.* 2014). Therefore, E-cadherin may affect mesoderm migration through modulation of FGF signaling and/or impairing the tissue’s mobility due to levels of adhesiveness.

Adhesion may also be impacted by CSPGs (Perez-Moreno *et al.* 2014). Our screen identified PTP99A, which is predicted to be a CSPG; the only other in *Drosophila* is Kon (Song *et al.* 2012). These CSPGs may regulate adhesion, like integrins, and/or FGF ligand-receptor interactions, like HSPGs. Kon is an ortholog of mammalian CSPG4 (Perez-Moreno *et al.* 2014) and shows defects in CVM migration (Figure 7K). Ptp99a shares sequence homology with the CSPG Phosphocan only across their cytoplasmic phosphatase regions. Ptp99a does not contain homology to the extracellular domain of Phosphocan, which comprises the CSPG. Nevertheless, overexpression of Ptp99a resulted in a moderate mesoderm phenotype (Figure S3K); however, whether this relates to CSPG activity is unclear but possible. In addition, identification of Cue through the screen is suggestive of the importance of trafficking of signaling components and/or adhesion molecules toward regulation of mesoderm development (Hirst and Carmichael 2011). The signaling pathways JAK/STAT and EGFR may also function in parallel with FGF to guide the spreading process.

Fourteen additional genes were identified (Table 1 and Figure S2). Although their weak endogenous expression and/or mild to no mutant spreading phenotype led us to conduct only a preliminary characterization, several genes are of note. Our screen isolated Toll-8, a receptor that has been reported to provide spatially localized heterophilic associations within the ectoderm necessary for supporting germband elongation (Pare *et al.* 2014). We also identified Toll-9, which is expressed in the mesoderm, and thus we hypothesize this Toll receptor may support a similar role in mesoderm development. Two enzymes were also uncovered, CG9550 and CG34056, which have the potential to function in the biosynthesis of heparan sulfate (HS) side chains found on HSPGs. Other enzymes of this class were previously found to impact mesoderm migration as they genetically interacted with FGFR Htl in this process (Lin *et al.* 1999). To address how HSPGs impact FGF signaling, in this study we decided to characterize the role of proteoglycans in supporting mesoderm migration because only limited information was available previously.

### Trol requirement in multiple pathways in *Drosophila*

Several studies have linked Trol with FGF signaling as well as other signaling pathways. While we highlight the role of Trol and Sdc in FGF signaling, our data also suggest that these HSPGs can modulate EGFR signaling as indicated by the decrease of dpERK in the tracheal pits of the ectoderm in mutant embryos (Figure 4K) (Gabay *et al.* 1997a) and also TGF-β signaling as revealed by cuticle defects (Figure 4O and Figure 5, I and M) (Ferguson and Anderson 1992). One of the earlier reports in *Drosophila* demonstrated that Trol is required for FGF signaling through the FGFR Breathless and FGF Branchless to support neuroblast proliferation (Park *et al.* 2003). They also showed that vertebrate Perlecan co-immunoprecipitated with vertebrate FGF-2 and that this interaction can be outcompeted upon addition of heparin. In addition, *trol* mutants displayed higher levels of Hedgehog (Hh), morphogen, nearer to its source of expression, suggesting that Trol is required for diffusion of Hh (Park *et al.* 2003). Another study yielded similar results in the neuromuscular junction by examining Wingless (Wg)-GFP of the Wnt pathway (Kamimura *et al.* 2013). Total Wg levels were not affected in *trol* mutants; however, Wg appeared to remain near the presynaptic membranes where it is secreted while the postsynaptic bouton acquired defects analogous to inhibition of Wnt signaling. These reports support the view that a general function for Trol is to effect ligand distribution.

### HSPG specificity in modulating different FGF-dependent processes

**Figure 8:**
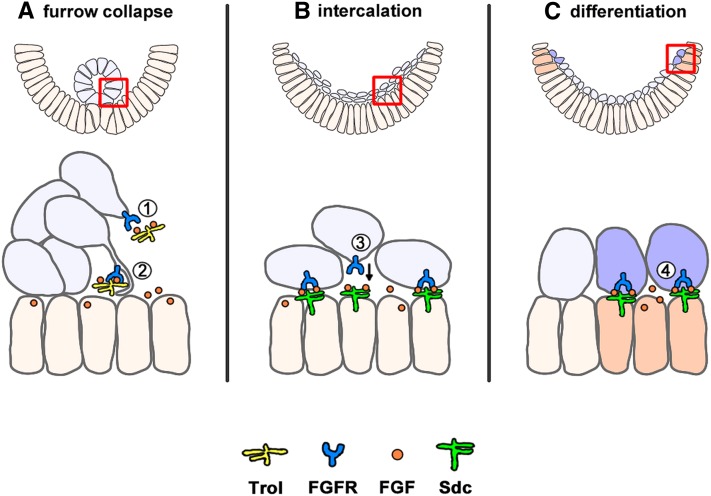
Model for differential action of Trol and Sdc HSPG-mediated activation of FGF signaling: hypothesized differences in signaling range. The mesoderm (blue) expresses the FGF receptor (FGFR) while the ectoderm (orange) expresses the FGF ligands. In mesoderm development, we show in this study that FGF signaling is modulated by Trol during the early steps of its migration (A) and by Sdc at later stages (B, C). Trol is secreted into the ECM and has the potential to signal to non-neighboring cells. This may occur through diffusion to target cells (#1) and/or the ability to be taken up by target cells via cytonemes (#2). Sdc is bound at the membrane and thus can only signal to adjacent cells to support small movements such as intercalation of the mesoderm (#3). Once migration is complete, Sdc can continue to act in neighboring FGF-producing cells (C, dark orange) for differentiation of cells at the dorsal mesoderm (C, dark blue) (#4).

Our screen isolated the HSPG Trol, a secreted protein, but another HSPG, Sdc, which contains a transmembrane domain, was reported previously to work with FGF during mesoderm development in the early embryo (Knox *et al.* 2011). Comparing Trol and Sdc revealed spatiotemporal differences in their expression (Figure 3, A and B and Figure 5, A–D) and nonoverlapping phenotypes relating to several FGF-dependent processes (Figure 4, Figure 5, Figure 6, and Figure 7). FGF signaling regulates a variety of activities that include communication between both distant cells and adjacent cells. However, their ability to modulate the range of FGF signaling is undetermined. Both Trol and Sdc are expressed in the ventral ectoderm during mesoderm migration (Figure 5, A and D arrows), and their expression patterns overlap with that of the FGF ligand Ths at these stages (Kadam *et al.* 2009). However, localized Trol is expressed earlier than Sdc. Furthermore, ectopic expression of Sdc in the mesoderm (both broadly and earlier than normal) results in a moderate spreading phenotype (Figure 5G), which we suggest is due to its sequestration of Ths ligand from Trol. Trol normally supports tube collapse, based on our genetic analysis, and likely is the only HSPG acting in this role. Based on these data, we propose the model that Trol, a component of the extracellular matrix (ECM), is able to promote FGF–FGFR interactions required for tube collapse in which mesoderm cells at a distance from the ectoderm respond to activation (“long-range” action). However, cell membrane–associated Sdc likely works locally to regulate FGF-FGFR interactions between neighboring cells (“short-range” action) as, for example, in the induction of dorsal somatic lineages (*e.g.*, Eve). As Trol is secreted, it may be better suited to support long-range or at least longer-range diffusion of the ligand relative to Sdc, which contains a transmembrane domain. For instance, during the FGF-dependent collapse of the invaginated tube of cells following EMT, Trol may aid in delivering FGF ligand to the receptor present in mesoderm cells located initially (before collapse) at a distance from the ectoderm (Figure 8A-1). Conversely, the fact that Sdc is membrane-associated suggests that Sdc, and not Trol, functions to support short-range FGF signaling in adjacent cells to support the processes of cell intercalation (Figure 8B) and cell differentiation (Figure 8C).

Another alternative hypothesis to that of diffusion is that Trol stabilizes FGF and allows presentation of the ligand to be taken up by cells expressing the receptor through cell protrusions such as cytonemes (Figure 8A-2) (Roy *et al.* 2014). These mechanisms may also play a role during dorsal mesoderm differentiation and CVM migration, both FGF-dependent processes, because Sdc is required for Eve specification while Trol is required in the CVM. Our model incorporates direct interaction between HSPGs and the FGF–FGFR complex, as supported by other studies (Pellegrini *et al.* 2000).

### HSPGs in ECM architecture

Alternatively, or in addition, it is possible that HSPGs affect receptor–ligand interactions indirectly by influencing distribution of the ligand through changes to organization of the basement membrane and ECM, which can result in positive or negative effects on signaling pathways (Kim *et al.* 2011). For example, S2R^+^ cell culture studies with the HSPG Dlp revealed that it can both enhance and inhibit Wnt signaling, depending on the context (Baeg *et al.* 2004). Recently, genetic interactions suggest that Trol sequesters the Ths ligand and prevents FGF-dependent differentiation in the larval lymph gland, thus serving an inhibitory role toward FGF signaling (Dragojlovic-Munther and Martinez-Agosto 2013). However, secreted HSPGs, such as Trol, are also components of the basement membrane and can modify organization of the ECM (Sarrazin *et al.* 2011). Perhaps in these lymph glands, Trol negatively regulates FGF signaling through changes to the ECM structure because the surrounding basement membrane was shown to also have defects that affected Hh distribution (Grigorian *et al.* 2013). Additionally, the ECM receptor Dystroglycan (Dg) has been shown to bind Trol and is found between the mesoderm–ectoderm interface (Schneider and Baumgartner 2008), thus potentially influencing Trol function during mesoderm migration. Therefore, *trol* mutants could also indirectly contribute to altered signaling activities, such as FGF distribution, at gastrulation due to changes in the ECM structure within these mutants.

### Extracellular *vs.* membrane-tethered HSPGs

In addition to Sdc function in late mesoderm specification (this study; Knox *et al.* 2011), several other reports support the view that membrane-bound HSPGs mediate short-range signaling. Axon guidance by Slit/Robo signaling in *Drosophila* embryos requires two HSPGs, Dlp and Sdc. The distribution and concentration of Dlp and Sdc are discrete to generate a distinct spatial field able to direct axonal growth (Smart *et al.* 2011). Another HSPG, Dally, is necessary in conjunction with BMP signaling for germline stem cell maintenance in *Drosophila* ovaries (Guo and Wang 2009). This requirement of Dally is limited to the germline only and not the nearby somatic cells, revealing its short range of action. In the vertebrate system, membrane-tethered HS chains are required for FGF signaling in adjacent cells during mouse embryogenesis (Shimokawa *et al.* 2011). All of these reports emphasize the importance of membrane-bound HSPGs in regulating ligand distribution and limiting signaling activity to a short distance. Alternatively, the property of Trol to be secreted is unique among *Drosophila* HSPGs. Our comparison of HSPGs Trol and Sdc in supporting FGF-dependent processes in the *Drosophila* early embryo has revealed that they support different signaling outputs. A future direction would be to examine whether their differential roles relate to how each HSPG affects FGF ligand distribution.

## Supplementary Material

Supporting Information
