# Supplementary material for: Ectopic Expression Screen Identifies Genes Affecting Drosophila Mesoderm Development Including the HSPG Trol
Source: G3 (Bethesda). 2014 Dec 23;5(2):301–13. doi: 10.1534/g3.114.015891 (PMC4321038; doi:10.1534/g3.114.015891)
Supplement: Supporting Information [file supp_5_2_301__index.html]

Ectopic Expression Screen Identifies Genes Affecting Drosophila Mesoderm Development Including the HSPG Trol — Supporting Information 

# Ectopic Expression Screen Identifies Genes Affecting *Drosophila* Mesoderm Development Including the HSPG Trol

## Supporting Information for Trisnadi and Stathopoulos, 2015

**Files in this Data Supplement:**

- Supporting Information - Figures S1-S3 and Table S1 (PDF, 747 KB)
- Figure S1 - Expressions and mutant phenotypes of genes identified in screen. (PDF, 450 KB)
- Figure S2 - Endogenous expression and mutant cross-sections of candidates identified from screen. (PDF, 272 KB)
- Figure S3 - Mesoderm phenotypes observed upon ectopic expression or reduction (RNAi) of additional HSPGs or a protein tyrosine phosphatase sharing homology with CSPGs. (PDF, 221 KB)
- Table S1 - Ectopic expression of twenty-four genes conferred lethality. (PDF, 141 KB)
