# Supplementary material for: Ectopic Expression Screen Identifies Genes Affecting Drosophila Mesoderm Development Including the HSPG Trol
Source: G3 (Bethesda). 2014 Dec 23;5(2):301–13. doi: 10.1534/g3.114.015891 (PMC4321038; doi:10.1534/g3.114.015891)
Supplement: Supporting Information [file supp_g3.114.015891_015891SI.pdf]

G3 · Genes | Genomes | Genetics

**Ectopic expression screen identifies genes affecting *Drosophila* mesoderm development including the HSPG Trol**

Nathanie Trisnadi[a] and Angelike Stathopoulos[a,*]

[a]Division of Biology and Biological Engineering, California Institute of Technology, 1200 East California Boulevard, MC 114-96, Pasadena, CA 91125, USA

[*]Corresponding author: angelike@caltech.edu; phone: 001-626-395-5855

**DOI: 10.1534/g3.114.015891**

**SUPPORTING INFORMATION**

**Figure S1:** **Expressions and mutant phenotypes of genes identified in screen.**

**Figure S2:** **Endogenous expression and mutant cross-sections of candidates identified from screen.**

**Figure S3:** **Mesoderm phenotypes when overexpressing or reducing additional HSPGs and CSPG.**

**Table S1:** **Ectopic expression of twenty-four genes conferred lethality.**

**FIGURE S1**

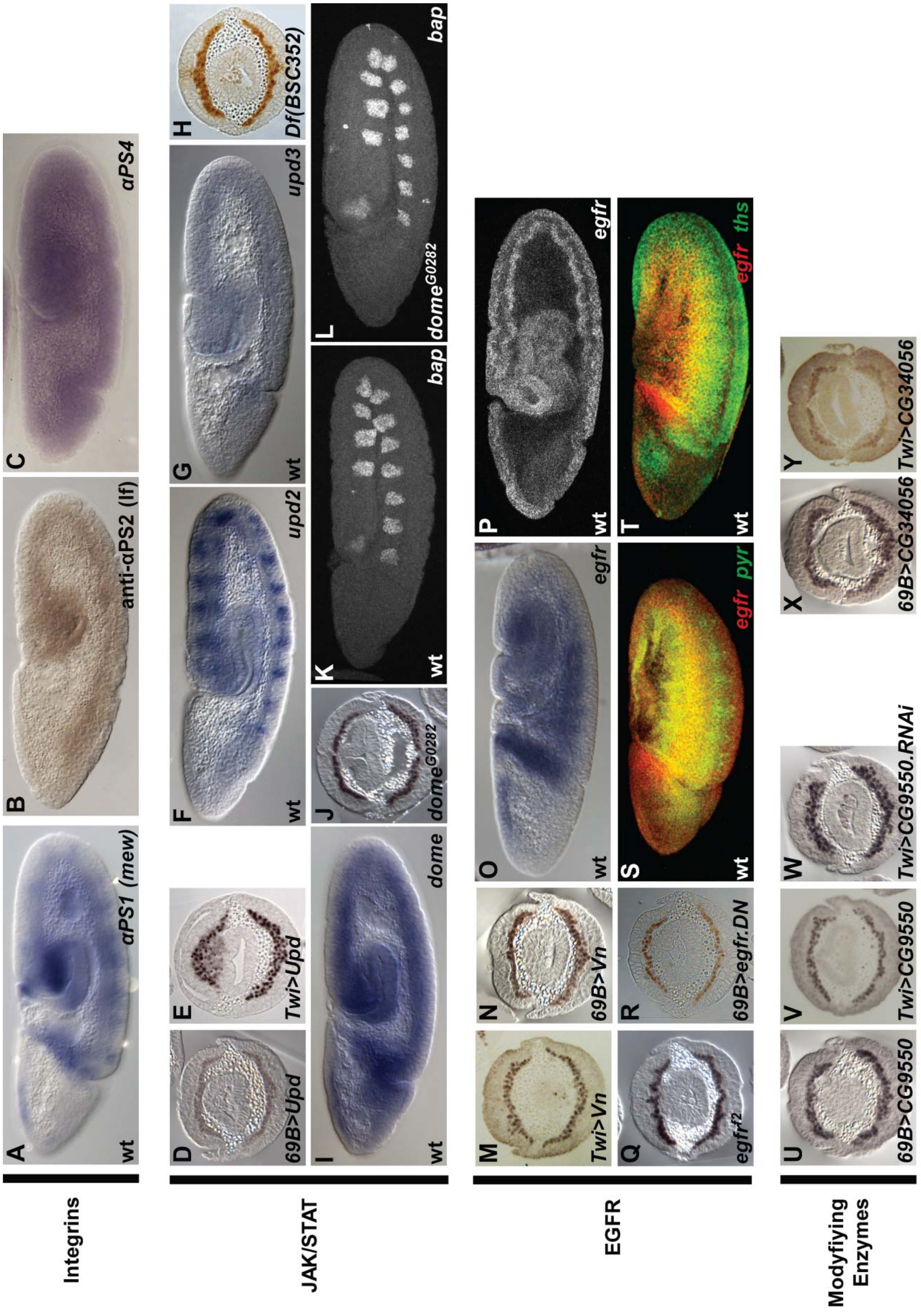

Integrins
JAK/STAT
EGFR
Modyfiying Enzymes
A
αPS1 (mew)
wt
B
anti-αPS2 (lf)
C
αPS4
D
69B>Upd
E
Twi>Upd
F
upd2
wt
G
upd3
wt
H
Df(BSC352)
I
dome
wt
J
dome$^{G0282}$
K
bap
wt
L
bap
dome$^{G0282}$
M
Twi>Vn
N
69B>Vn
O
egfr
wt
P
egfr
wt
Q
egfr$^{f2}$
R
69B>egfr.DN
S
egfr pyr
wt
T
egfr ths
wt
U
69B>CG9550
V
Twi>CG9550
W
Twi>CG9550.RNAi
X
69B>CG34056
Y
Twi>CG34056

**Figure S1. Expressions and mutant phenotypes of genes identified in screen.**

In situ hybridization was performed using riboprobes against the indicated genes. Lateral views of whole mount embryos are positioned with anterior facing left and dorsal side facing up. Cross-sectioned stage 10 embryos were stained with α-Twist to mark mesoderm cells.

Integrins: Wildtype expression patterns for (A) α-PS1, (B) α-PS2 and (C) α-PS4 show integrins are present at stage 8 embryos. α-PS2 (Inflated) was specifically found to be upregulated in the mesoderm (Y-K.Bae and A.S., unpub. obs.)

JAK/STAT: Cross-section of embryos overexpressing Upd in the (D) ectoderm and (E) mesoderm reveal multilayer phenotype. RNA expression of ligands (F) *upd2* and (G) *upd3* in wildtype embryos. (H) Cross-section of deficiency covering all three *upd* ligands has a mild spreading phenotype. (I) Wildtype expression of receptor *dome* shows upregulation in the mesoderm. Dome was also identified in a separate screen of mesoderm factors (Y-K.Bae and A.S., unpub. obs.). (J) Cross-section of *dome* mutant embryos have wildtype spreading. *bap* expression (Azpiazu and Frasch 1993) in (K) wildtype is comparable to (L) *dome* mutant embryos, indicating normal mesoderm spreading.

EGFR: Cross-section of embryos overexpressing Vn in the (M) mesoderm and (N) ectoderm. Wildtype (O) stage 7 and (P) stage 10 embryos reveals *egfr* switch from ectodermal to mesodermal expression. Cross-section of embryos (Q) mutant for *egfr* or (R) overexpressing the dominant negative form of *egfr* in the ectoderm have relatively normal spreading. Wildtype expression of *egfr* and FGF ligands (S) *pyr* and (T) *ths* show overlapping domains at stage 7, suggesting a possibility of EGFR affecting FGF ligands.

Modifying enzymes: Cross-section of embryos overexpressing CG9550 in the ectoderm have spreading defects (U), while overexpression in the mesoderm is normal (V). (W) Embryos removing *cg9550* by RNAi in the mesoderm results in a multilayer. Together, these date suggest a role for CG9550 in the mesoderm. Similarly, cross-sections of embryos overexpressing CG34056 in the ectoderm (X), but not mesoderm (Y), show spreading defects.

**FIGURE S2**

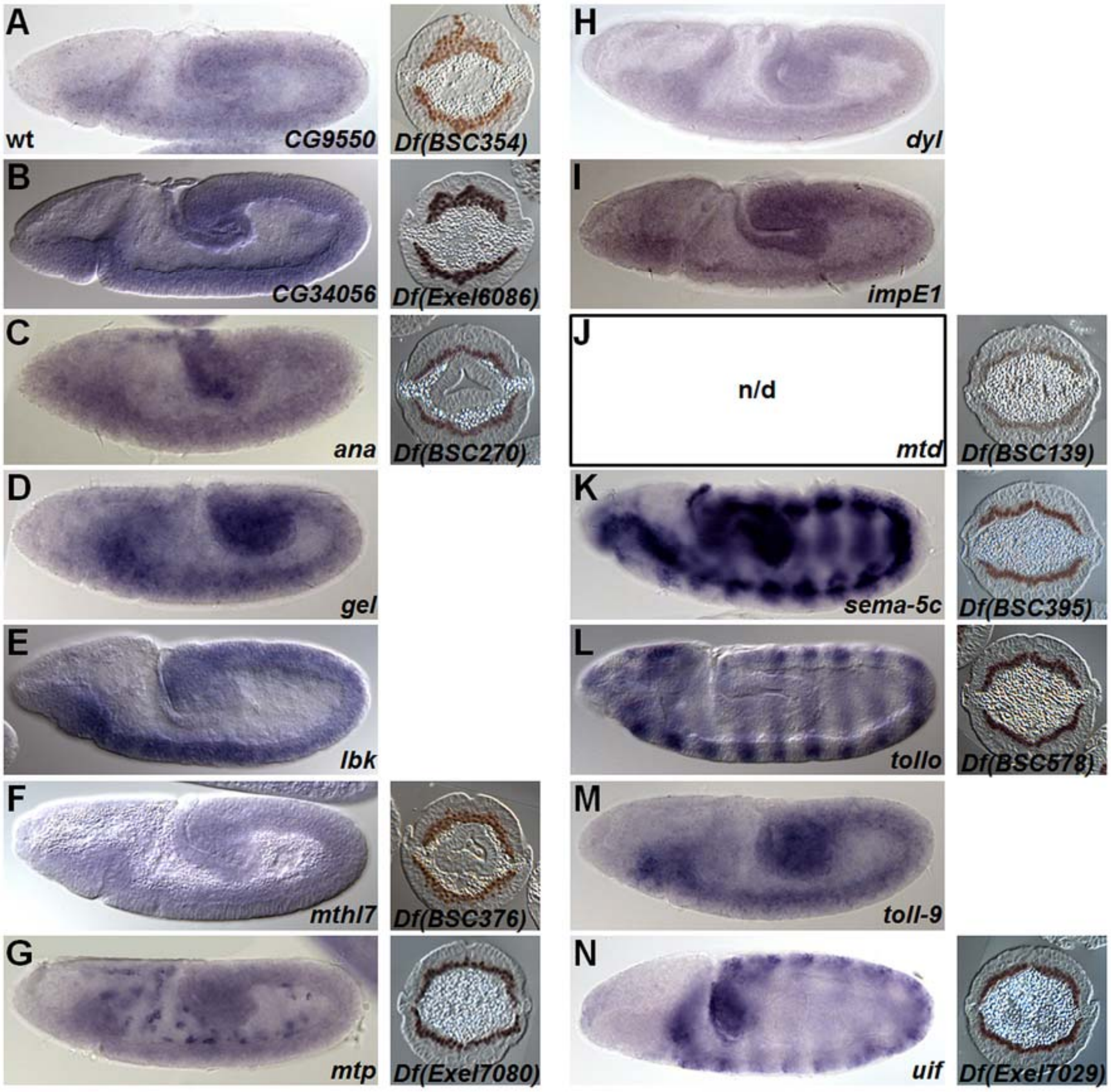


**Figure S2. Endogenous expression and mutant cross-sections of candidates identified from screen.**

For each indicated gene, endogenous expression was determined by in situ hybridization on whole mount *yw* embryos. Deficiencies uncovering the gene were stained with anti-Twist and sectioned to ascertain if mesoderm migration was affected. The genes examined are (A) *CG9550*, (B) *CG34056* (C) *anachronism*, (D) *gelsolin*, (E) *lambik*, (F) *methuselah-like 7*, (G) *microsomal triacylglycerol transfer protein*, (H) *dusky-like*, (I) *ecdysone-inducible gene E1*, (J) *i(3)82Fd/mustard*, (K) *semaphorin-5c*, (L) *toll-8*, (M) *toll -9*, and (N) *uninflatable*. Two modifying enzymes, sulfotransferase (A) and galactosyltransferase (B) gave the most severe spreading phenotype. Although their corresponding UAS insertion was verified (data not show, see Materials and Methods), their endogenous mesoderm expression appeared weak. See Table 1, Table 2, and Table S1 for more information.

**FIGURE S3**

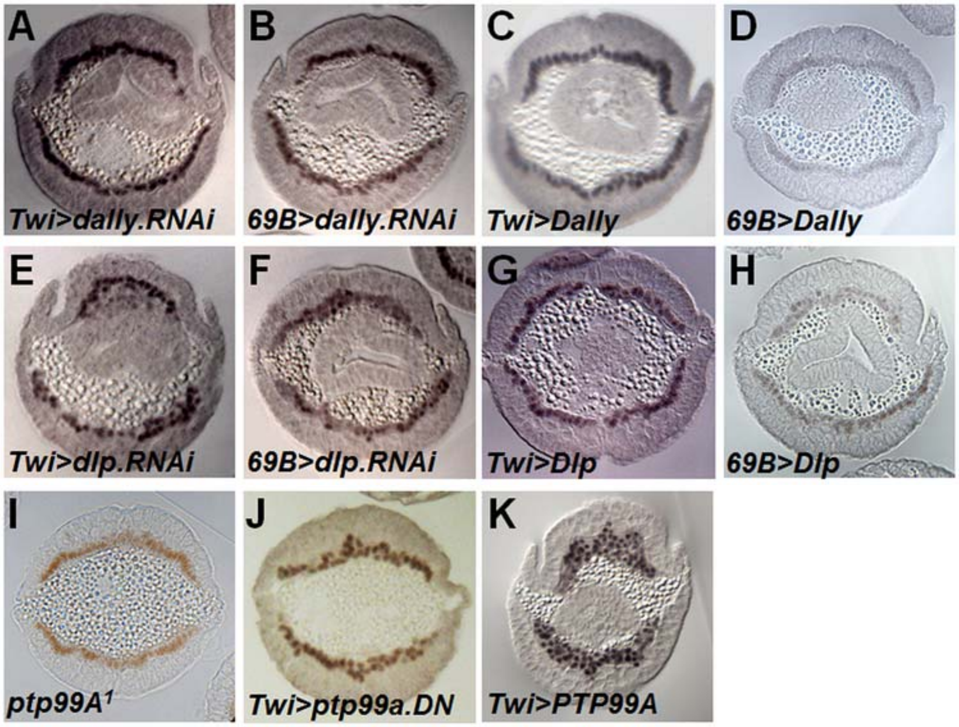


**Figure S3. Mesoderm phenotypes observed upon ectopic expression or reduction (RNAi) of additional HSPGs or a protein tyrosine phosphatase sharing homology with CSPGs.**

RNAi and ectopic expression mutant analysis for HSPGs Dally (A-D) and Dally-like (E-H), and CHSPG Ptp99a (I-K) revealed mild to no effects on mesoderm spreading. (J) DN refers to the dominant negative form of *ptp99a* in which the phosphatase domain is deleted (see Table S1).

| Gene ID | Name | | UAS | Lethality | Localization | Mutant allele(s) | Function |
|---|---|---|---|---|---|---|---|
| CG8095 | α-PS3/Scab | Scb | EP 2591 | Twi-Gal4 | cell surface | scab$^{2}$ | alpha-PS3 integrin |
| CG5372 | α-PS5 | IntαPS5 | GS 12413 | 69B-Gal4 | cell surface | αPS5$^{[MI01533]}$ | integrin-related |
| CG8084 | Anachronism | Ana | GS 9498 | Twi-Gal4 | secreted | Df(2R)BSC270 | growth factor activity |
| CG4531 | Argos | Aos | GS 12984 | 69B-Gal4 | secreted | Df(3L)BSC562 | antagonist of EGFR signalling |
| CG12086 | Cueball | Cue | EY 1263 | 69B-Gal4 | cell surface | cue$^{2}$ - hypomorph | LDLR class B repeats; EGF-like |
| CG15013 | Dusky-like | Dyl | GS 20894 | 69B-Gal4 | cell surface | | zona pellucida domain |
| CG3722 | E-cadherin/ Shotgun | Shg | XP d01606 | 69B-Gal4 | cell surface | shg$^{2}$ - amorph | cadherin |
| CG32356 | Ecdysone-inducible gene E1 | ImpE1 | GS 11510 | 69B-Gal4 | cell surface | | LDLR class A repeat |
| CG1106 | Gelsolin | Gel | GS 10156 | Twi-Gal4 | secreted | | actin binding |
| CG32464 | l(3)82Fd/ Mustard | Mtd | GS 16948 | 69B-Gal4 | secreted | Df(3R)BSC139 | peptidoglycan-binding LysM domain |
| CG8434 | Lambik | Lbk | GS 17119 | Twi-Gal4 | cell surface | | cell adhesion, Ig domain, LRR |
| CG7476 | Methuselah-like 7 | Mthl7 | GS 21256 | Twi-Gal4 | cell surface | Df(3L)BSC376 | GPCR |
| CG9342 | Microsomal triacylglycerol transfer protein | Mtp | XP d07488 | Twi-Gal4 | secreted | Df(2L)Exel7080 | lipid transport protein; triglyceride binding |
| CG2005 | Protein tyrosine phosphatase 99A | Ptp99A | EY 7423 | Twi-Gal4 | cell surface | ptp99a$^{1}$ - phosphatase domain delete; UAS-ptp99a.DN (K. Zinn) | Fibronectin type III domain |
| CG13194 | Pyramus | Pyr | GS 22603 | 69B-Gal4 | secreted | pyr$^{e02915}$; pyr$^{18}$; Df(2R)BSC25 | FGF ligand |
| CG5661 | Semaphorin-5c | Sema-5c | EY 1704 | 69B-Gal4 | cell surface | Df(3L)BSC395 | Plexin; Sema domain |
| CG33950 | Terribly reduced optic lobes | Trol | GE 10067 | 69B-Gal4 | secreted | trol$^{G0211}$, FRT19A *111801; UAS-trol.RNAi §24549 | HSPG; Perlecan |
| CG6890 | Toll-8 | Tollo | XP d01565 | 69B-Gal4 | cell surface | Df(3L)BSC578 | LLR; Toll/interleukin-1 receptor homology (TIR) domain |
| CG5528 | Toll-9 | Toll-9 | GS 51 | 69B-Gal4 | cell surface | | LLR; Toll/interleukin-1 receptor homology (TIR) domain |
| CG9138 | Uninflatable | Uif | GS 11655 | 69B-Gal4 | cell surface | Df(2L)Exel7029 | LDLR class A repeat; EGF-like calium binding |
| CG5993 | Unpaired/ Outstretched | Upd/Os | G17133 | Twi-Gal4 & 69B-Gal4 | secreted | upd$^{4}$ - loss of fxn; Df(1)BSC352 | JAK/STAT ligand |
| CG10491 | Vein | Vn | GS 12044 | Twi-Gal4 & 69B-Gal4 | secreted | vn$^{C221}$ | EGFR ligand |
| CG34056 | galactosyltransferase | | GS 11028 | 69B-Gal4 | cell surface | Df(3L)Exel6086 | beta-1,3-galactosyl-transferase activity |
| CG9550 | sulfotransferase | | GS 18034 | 69B-Gal4 | cell surface | Df(2L)BSC354; UAS-RNAi §100976 | chondroitin 6-sulfotransferase activity |
| ***Additional genes used in this study*** | | | | | | | |
| CG14226 | Domeless | Dome | | | cell surface | dome$^{G0282}$ - loss of fxn | JAK/STAT receptor |
| CG10079 | EGFR/ Torpedo | | | | cell surface | egfr$^{f2}$; UAS-egfr.DN #5364 | EGF Receptor |
| CG10497 | Syndecan | Sdc | | | cell surface | sdc$^{2639}$, FRT42B (M. Freeman); UAS-Sdc #8564; UAS-sdc.RNAi §13322 | HSPG |
| CG4974 | Dally | | | | cell surface | UAS-Dally #5397; UAS-dally.RNAi §14136 | HSPG |
| CG32146 | Dally-like | Dlp | | | cell surface | UAS-Dlp #9160; UAS-dlp.RNAi §10299 | HSPG |
| CG10275 | Kon-tiki/ Perdido | Kon/ Perd | | | cell surface | UAS-kon.RNAi §37283 | CSPG |

**#  Bloomington Stock Center**
**§  VDRC Stock Center**
***  DGRC Kyoto Stock Center**

**Table S1. Ectopic expression of twenty-four genes conferred lethality.**

Complete list of the twenty-four genes that resulted in lethality when overexpressed with Twi-Gal4 and/or 69B-Gal4. The genotypes used in this study and their predicted/known functions are also listed here. Genes in red indicate those with mesoderm spreading defects and/or relevant expression patterns and were further analyzed. Pyramus in blue has previously been well characterized. Additional genes that were examined in this study are noted as well.
