# Supplementary material for: Ectopic Expression Screen Identifies Genes Affecting Drosophila Mesoderm Development Including the HSPG Trol
Source: G3 (Bethesda). 2014 Dec 23;5(2):301–13. doi: 10.1534/g3.114.015891 (PMC4321038; doi:10.1534/g3.114.015891)
Supplement: Supporting Information [file supp_g3.114.015891_FigureS1.pdf]

FIGURE S1

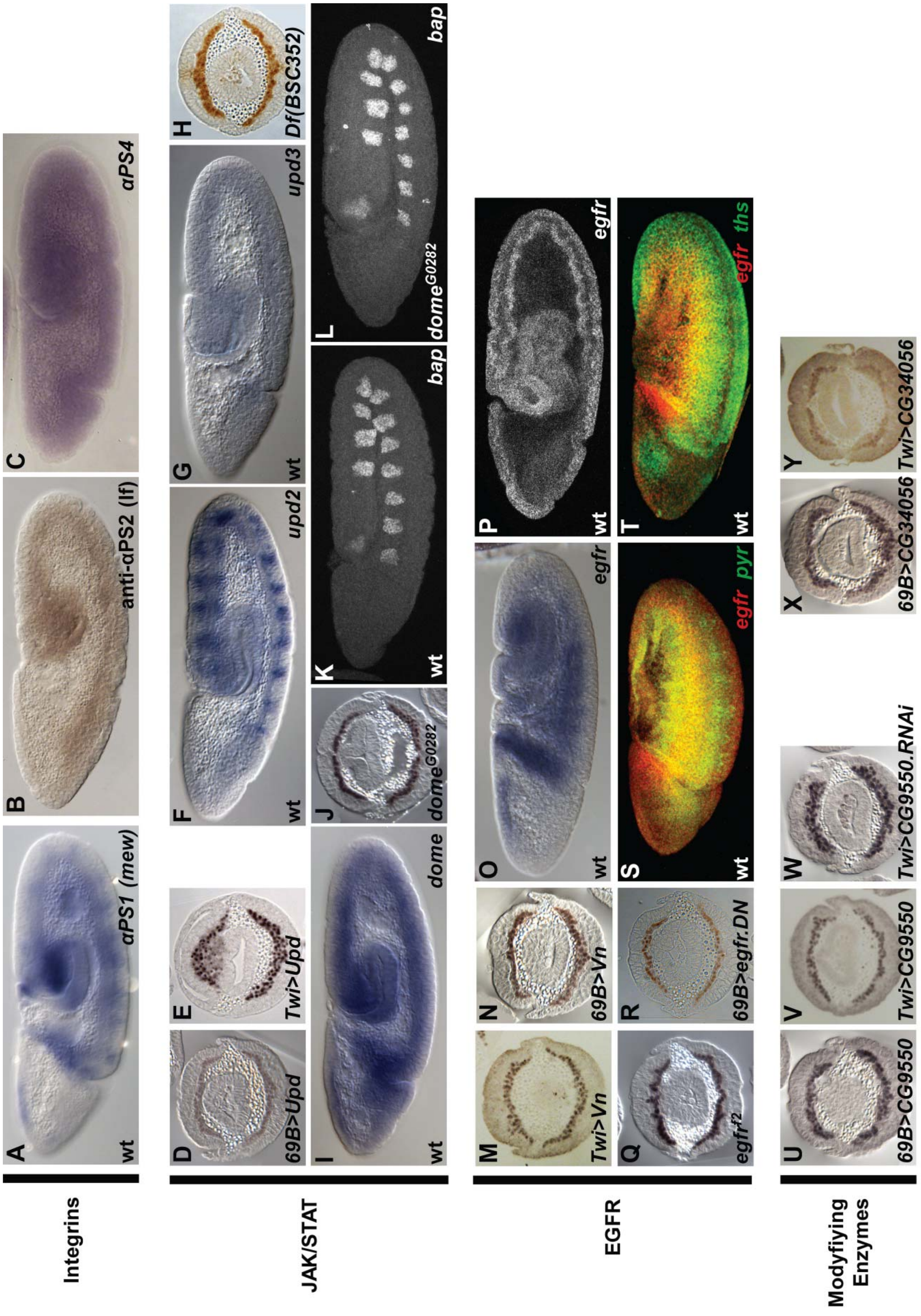

Integrins
JAK/STAT
EGFR
Modyfiying Enzymes
A wt αPS1 (mew)
B anti-αPS2 (lf)
C αPS4
D 69B>Upd
E Twi>Upd
F wt upd2
G wt upd3
H Df(BSC352)
I wt dome
J dome^{G0282}
K wt bap
L dome^{G0282} bap
M Twi>Vn
N 69B>Vn
O wt egfr
P wt egfr
Q egfr^{f2}
R 69B>egfr.DN
S wt egfr pyr
T wt egfr ths
U 69B>CG9550
V Twi>CG9550
W Twi>CG9550.RNAi
X 69B>CG34056
Y Twi>CG34056

Figure S1. Expressions and mutant phenotypes of genes identified in screen.

In situ hybridization was performed using riboprobes against the indicated genes. Lateral views of whole mount embryos are positioned with anterior facing left and dorsal side facing up. Cross-sectioned stage 10 embryos were stained with α-Twist to mark mesoderm cells.

Integrins: Wildtype expression patterns for (A) α-PS1, (B) α-PS2 and (C) α-PS4 show integrins are present at stage 8 embryos. α-PS2 (Inflated) was specifically found to be upregulated in the mesoderm (Y-K.Bae and A.S., unpub. obs.)

JAK/STAT: Cross-section of embryos overexpressing Upd in the (D) ectoderm and (E) mesoderm reveal multilayer phenotype. RNA expression of ligands (F) *upd2* and (G) *upd3* in wildtype embryos. (H) Cross-section of deficiency covering all three *upd* ligands has a mild spreading phenotype. (I) Wildtype expression of receptor *dome* shows upregulation in the mesoderm. Dome was also identified in a separate screen of mesoderm factors (Y-K.Bae and A.S., unpub. obs.). (J) Cross-section of *dome* mutant embryos have wildtype spreading. *bap* expression (Azpiazu and Frasch 1993) in (K) wildtype is comparable to (L) *dome* mutant embryos, indicating normal mesoderm spreading.

EGFR: Cross-section of embryos overexpressing Vn in the (M) mesoderm and (N) ectoderm. Wildtype (O) stage 7 and (P) stage 10 embryos reveals *egfr* switch from ectodermal to mesodermal expression. Cross-section of embryos (Q) mutant for *egfr* or (R) overexpressing the dominant negative form of *egfr* in the ectoderm have relatively normal spreading. Wildtype expression of *egfr* and FGF ligands (S) *pyr* and (T) *ths* show overlapping domains at stage 7, suggesting a possibility of EGFR affecting FGF ligands.

Modifying enzymes: Cross-section of embryos overexpressing CG9550 in the ectoderm have spreading defects (U), while overexpression in the mesoderm is normal (V). (W) Embryos removing *cg9550* by RNAi in the mesoderm results in a multilayer. Together, these date suggest a role for CG9550 in the mesoderm. Similarly, cross-sections of embryos overexpressing CG34056 in the ectoderm (X), but not mesoderm (Y), show spreading defects.
