# Supplementary material for: Ectopic Expression Screen Identifies Genes Affecting Drosophila Mesoderm Development Including the HSPG Trol
Source: G3 (Bethesda). 2014 Dec 23;5(2):301–13. doi: 10.1534/g3.114.015891 (PMC4321038; doi:10.1534/g3.114.015891)
Supplement: Supporting Information [file supp_g3.114.015891_FigureS2.pdf]

FIGURE S2

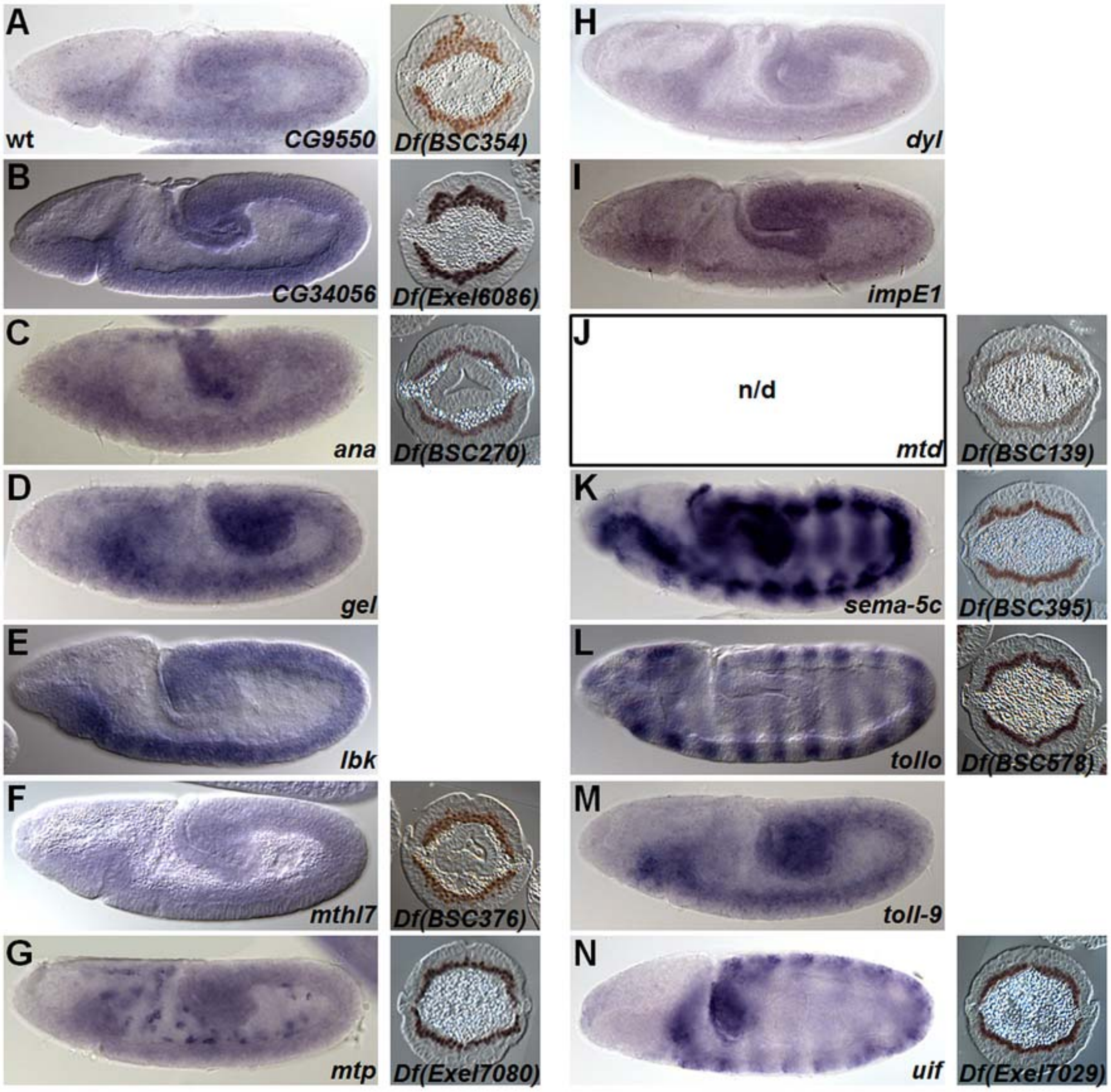

Figure S2. Endogenous expression and mutant cross-sections of candidates identified from screen.

For each indicated gene, endogenous expression was determined by in situ hybridization on whole mount *yw* embryos. Deficiencies uncovering the gene were stained with anti-Twist and sectioned to ascertain if mesoderm migration was affected. The genes examined are (A) *CG9550*, (B) *CG34056* (C) *anachronism*, (D) *gelsolin*, (E) *lambik*, (F) *methuselah-like 7*, (G) *microsomal triacylglycerol transfer protein*, (H) *dusky-like*, (I) *ecdysone-inducible gene E1*, (J) *i(3)82Fd/mustard*, (K) *semaphorin-5c*, (L) *toll-8*, (M) *toll -9*, and (N) *uninflatable*. Two modifying enzymes, sulfotransferase (A) and galactosyltransferase (B) gave the most severe spreading phenotype. Although their corresponding UAS insertion was verified (data not show, see Materials and Methods), their endogenous mesoderm expression appeared weak. See Table 1, Table 2, and Table S1 for more information.
