# Supplementary material for: Ectopic Expression Screen Identifies Genes Affecting Drosophila Mesoderm Development Including the HSPG Trol
Source: G3 (Bethesda). 2014 Dec 23;5(2):301–13. doi: 10.1534/g3.114.015891 (PMC4321038; doi:10.1534/g3.114.015891)
Supplement: Supporting Information [file supp_g3.114.015891_FigureS3.pdf]

FIGURE S3

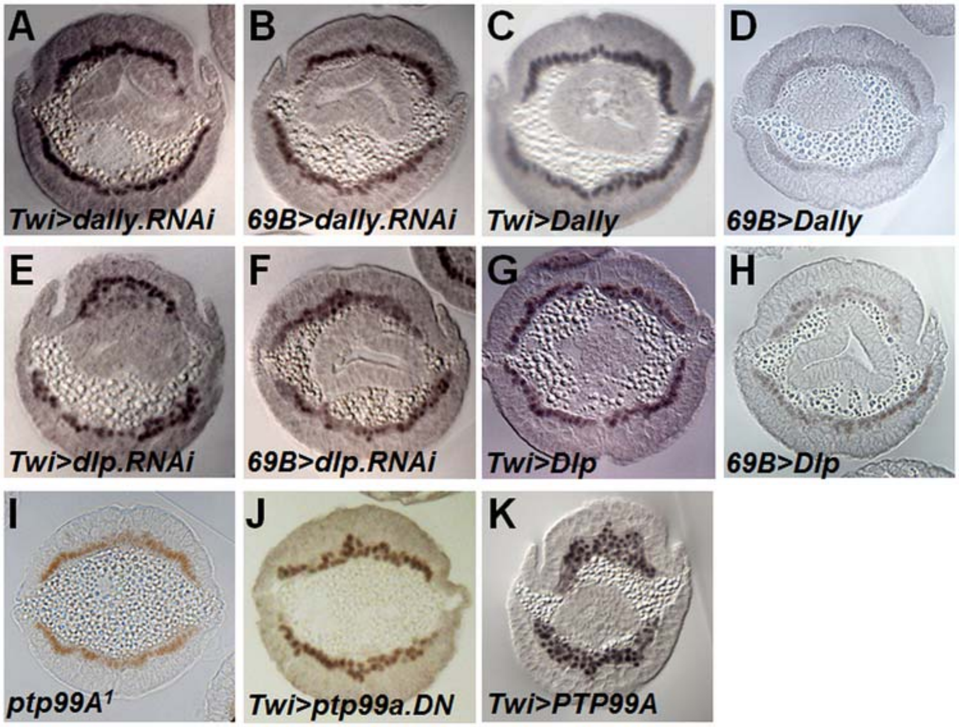

Figure S3. Mesoderm phenotypes observed upon ectopic expression or reduction (RNAi) of additional HSPGs or a protein tyrosine phosphatase sharing homology with CSPGs.

RNAi and ectopic expression mutant analysis for HSPGs Dally (A-D) and Dally-like (E-H), and CHSPG Ptp99a (I-K) revealed mild to no effects on mesoderm spreading. (J) DN refers to the dominant negative form of *ptp99a* in which the phosphatase domain is deleted (see Table S1).
