# Supplementary material for: Ectopic Expression Screen Identifies Genes Affecting Drosophila Mesoderm Development Including the HSPG Trol
Source: G3 (Bethesda). 2014 Dec 23;5(2):301–13. doi: 10.1534/g3.114.015891 (PMC4321038; doi:10.1534/g3.114.015891)
Supplement: Supporting Information [file supp_g3.114.015891_TableS1.pdf]

Gene ID	Name		UAS	Lethality	Localization	Mutant allele(s)	Function
CG8095	α-PS3/Scab	Scb	EP 2591	Twi-Gal4	cell surface	$scab^{2}$	alpha-PS3 integrin
CG5372	α-PS5	IntαPS5	GS 12413	69B-Gal4	cell surface	$\alpha PS5^{[MI01533]}$	integrin-related
CG8084	Anachronism	Ana	GS 9498	Twi-Gal4	secreted	Df(2R)BSC270	growth factor activity
CG4531	Argos	Aos	GS 12984	69B-Gal4	secreted	Df(3L)BSC562	antagonist of EGFR signalling
CG12086	Cueball	Cue	EY 1263	69B-Gal4	cell surface	cue^{2} - hypomorph	LDLR class B repeats; EGF-like
CG15013	Dusky-like	Dyl	GS 20894	69B-Gal4	cell surface		zona pellucida domain
CG3722	E-cadherin/ Shotgun	Shg	XP d01606	69B-Gal4	cell surface	shg^{2} - amorph	cadherin
CG32356	Ecdysone-inducible gene E1	ImpE1	GS 11510	69B-Gal4	cell surface		LDLR class A repeat
CG1106	Gelsolin	Gel	GS 10156	Twi-Gal4	secreted		actin binding
CG32464	l(3)82Fd/ Mustard	Mtd	GS 16948	69B-Gal4	secreted	Df(3R)BSC139	peptidoglycan-binding LysM domain
CG8434	Lambik	Lbk	GS 17119	Twi-Gal4	cell surface		cell adhesion, Ig domain, LRR
CG7476	Methuselah-like 7	Mthl7	GS 21256	Twi-Gal4	cell surface	Df(3L)BSC376	GPCR
CG9342	Microsomal triacylglycerol transfer protein	Mtp	XP d07488	Twi-Gal4	secreted	Df(2L)Exel7080	lipid transport protein; triglyceride binding
CG2005	Protein tyrosine phosphatase 99A	Ptp99A	EY 7423	Twi-Gal4	cell surface	$ptp99a^{1}$ - phosphatase domain delete; UAS-ptp99a.DN (K. Zinn)	Fibronectin type III domain
CG13194	Pyramus	Pyr	GS 22603	69B-Gal4	secreted	pyr^{e02915}; pyr^{18}; Df(2R)BSC25	FGF ligand
CG5661	Semaphorin-5c	Sema-5c	EY 1704	69B-Gal4	cell surface	Df(3L)BSC395	Plexin; Sema domain
CG33950	Terribly reduced optic lobes	Trol	GE 10067	69B-Gal4	secreted	$trol^{G0211}$, FRT19A *111801; UAS-trol.RNAi §24549	HSPG; Perlecan
CG6890	Toll-8	Tollo	XP d01565	69B-Gal4	cell surface	Df(3L)BSC578	LLR; Toll/interleukin-1 receptor homology (TIR) domain
CG5528	Toll-9	Toll-9	GS 51	69B-Gal4	cell surface		LLR; Toll/interleukin-1 receptor homology (TIR) domain
CG9138	Uninflatable	Uif	GS 11655	69B-Gal4	cell surface	Df(2L)Exel7029	LDLR class A repeat; EGF-like calium binding
CG5993	Unpaired/ Outstretched	Upd/Os	G17133	Twi-Gal4 & 69B-Gal4	secreted	upd^{4} - loss of fxn; Df(1)BSC352	JAK/STAT ligand
CG10491	Vein	Vn	GS 12044	Twi-Gal4 & 69B-Gal4	secreted	vn^{C221}	EGFR ligand
CG34056	galactosyltransferase		GS 11028	69B-Gal4	cell surface	Df(3L)Exel6086	beta-1,3-galactosyl-transferase activity
CG9550	sulfotransferase		GS 18034	69B-Gal4	cell surface	Df(2L)BSC354; UAS-RNAi §100976	chondroitin 6-sulfotransferase activity
Additional genes used in this study							
CG14226	Domeless	Dome			cell surface	$dome^{G0282}$ - loss of fxn	JAK/STAT receptor
CG10079	EGFR/ Torpedo				cell surface	$egfr^{f2}$; UAS-egfr.DN #5364	EGF Receptor
CG10497	Syndecan	Sdc			cell surface	sdc^{2639}, FRT42B (M. Freeman); UAS-Sdc #8564; UAS-sdc.RNAi §13322	HSPG
CG4974	Dally				cell surface	UAS-Dally #5397; UAS-dally.RNAi §14136	HSPG
CG32146	Dally-like	Dlp			cell surface	UAS-Dlp #9160; UAS-dlp.RNAi §10299	HSPG
CG10275	Kon-tiki/ Perdido	Kon/ Perd			cell surface	UAS-kon.RNAi §37283	CSPG

# Bloomington Stock Center
§ VDRC Stock Center
*** DGRC Kyoto Stock Center**

Table S1. Ectopic expression of twenty-four genes conferred lethality.

Complete list of the twenty-four genes that resulted in lethality when overexpressed with Twi-Gal4 and/or 69B-Gal4. The genotypes used in this study and their predicted/known functions are also listed here. Genes in red indicate those with mesoderm spreading defects and/or relevant expression patterns and were further analyzed. Pyramus in blue has previously been well characterized. Additional genes that were examined in this study are noted as well.
